# The prominent alteration in transcriptome and metabolome of *Mycobacterium bovis* BCG str. Tokyo 172 induced by vitamin B_1_

**DOI:** 10.1186/s12866-019-1492-9

**Published:** 2019-05-22

**Authors:** Ningning Song, Zhaoli Li, Ziyin Cui, Liping Chen, Yingying Cui, Guanghui Dang, Zhe Li, He Li, Siguo Liu

**Affiliations:** State Key Laboratory of Veterinary Biotechnology, Harbin Veterinary Research Institute, Chinese Academy of Agricultural Sciences, Harbin, China

**Keywords:** Transcriptomics, Metabolomics, BCG, Vitamin B_1_, Growth inhibition

## Abstract

**Background:**

Vitamin B_1_ (V_B1_) is a crucial dietary nutrient and essential cofactor for several key enzymes in the regulation of cellular and metabolic processes, and more importantly in the activation of immune system. To date, the precise role of V_B1_ in *Mycobacterium tuberculosis* remains to be fully understood.

**Results:**

In this study, the transcriptional and metabolic profiles of V_B1_-treated *Mycobacterium. bovis* BCG were analyzed by RNA-sequencing and LC-MS (Liquid chromatography coupled to mass spectrometry). The selection of BCG strain was based on its common physiological features shared with *M. tuberculosis*. The results of cell growth assays demonstrated that V_B1_ inhibited the BCG growth rate in vitro. Transcriptomic analysis revealed that the expression levels of genes related to fatty acid metabolism, cholesterol metabolism, glycolipid catabolism, DNA replication, protein translation, cell division and cell wall formation were significantly downregulated in *M. bovis* BCG treated with V_B1._ In addition, the metabolomics LC-MS data indicated that most of the amino acids and adenosine diphosphate (ADP) were decreased in *M. bovis* BCG strain after V_B1_ treatment.

**Conclusions:**

This study provides the molecular and metabolic bases to understand the impacts of V_B1_ on *M.bovis BCG*.

**Electronic supplementary material:**

The online version of this article (10.1186/s12866-019-1492-9) contains supplementary material, which is available to authorized users.

## Background

*Mycobacterium tuberculosis* (Mtb), causative agent for Tuberculosis, is the leading infectious cause of death worldwide. The difficulties associated with the treatment and control of tuberculosis are mainly due to the ability of Mtb to persist in a dormant state and maintain viability in the absence of cellular replication. Although the use of anti-TB drugs such as rifampicin (RIF) and isoniazid (INH) has been widely accepted, the treatment outcome may be worsened by the presence of multidrug resistant (MDR) strains of Mtb. Moreover, the appearance of MDR and XDR (extensively drug resistant) strains can reduce the treatment success in TB. Therefore, the discovery of novel anti-Tuberculosis drugs and the implementation of effective Tuberculosis prevention programme have become a major focus of Tuberculosis research.

The application of transcriptomics has been driven by bioinformatic analysis for the identification of key variable genes that upregulated and downregulated in bacterial strains under different conditions. The primary aim of this approach is to decipher how the pathogens regulate their gene expression and host transcriptional machinery. This approach will provide a better understanding of molecular events and help to identify the key genes responsible for the pathogenesis of Mtb under different exposure conditions. For instance, transcriptional studies have been applied in Mtb under nutrient starvation, acidic and oxidative stress conditions [1–3]. Moreover, transcriptional profiling have been carried out on soil bacterium *M. smegmatis* and Mtb following the exposure to low and high levels of hydrogen peroxide and to vitamin C (Vc), respectively [4, 5]. In vivo*,* macrophages with similar host environment have been used to study the host response to infection [5]. Numerous transcriptional studies have been conducted using primary cultures of human and murine macrophages [6–8].

Metabolomics has been used to describe the complete set of complicated and interrelated chemical transformations that enable individual cells to replicate and survive. Metabolite represents the final downstream outcome of genome transcription, which contains a mixture of high- and low-molecular weight compounds involved in the metabolic reactions during normal cell growth and preservation [9]. Due to the importance of metabolism, numerous studies have been focused on Mtb metabolism, including central carbon metabolism [10], cofactor metabolism [11, 12], sulfur, nitrogen and phosphorus metabolism [13, 14]. More importantly, metabolomic analysis enables us to identify the potential biomarkers for diseases. For instance, the impact of Mtb infection on host metabolism has been studied in several experimental models such as mice and guinea pigs [15, 16]. Moreover, clinical subjects with distinct metabolite profiles have been used to distinguish uninfected patients from those with active disease and latent infection.

Thiamin (Vitamin B_1_), in its active form thiamin diphosphate (ThDP), is an essential cofactor for all organisms [17–19]. Vitamin B_1_ (V_B1_) is involved in energy metabolism and the degradation of sugars and carbon skeleton [19]. V_B1_ has a multifaceted role in the regulation of gut immunity by maintaining the functions of naive B cells and utilizes the energy released from the citric acid cycle [20]. In addition, V_B1_ participates in the activation of immune system, nerve tissue repair, neuronal communication, brain development, brain function and cell-membrane dynamics [21–23]. Furthermore, V_B1_ can diminish the concentrations of reactive nitrogen species in cells as a consequence of its antioxidant activity, but has no significant effects on reactive oxygen species [24]. Despite the fact that bacteria, fungi and plants are able to synthesize V_B1_ de novo, mammals are entirely reliant on a dietary source of V_B1_. Nevertheless, V_B1_ deficiency may result in an increased lactic acid production and lead to metabolic and neurological disorders [25].

To date, vitamin A (V_A_), V_C_ and vitamin D (V_D_) supplementation have been used as adjunct to anti-tuberculosis drugs [26–28]. V_C_ is capable of inhibiting the growth of Mtb due to the reduction of ferric to ferrous ions. The ferrous ions may result in the production of reactive oxygen species (i.e. hydrogen peroxide, superoxide and hydroxyl radicals) through Harber–Weiss cycle and Fenton reactions, which give rise to pleiotropic effects associated with certain cellular processes [28], V_D_ itself may not exert anti-TB activity; instead, its metabolite 1,25(OH)_2_D_3_ (1α,25-dihydroxyvitamin D_3_) can inhibit the growth of Mtb and regulate the immunity of host cells via phagosome-lysosome fusion [29]. Additionally, V_B5_ can reduce the growth of intracellular Mtb in macrophage in vitro, and confer protection against mycobacterial infection by enhancing the maturity of macrophages and inducing the differentiation of Th1 and Th17 cells in mice infected with Mtb [30]. Our recent study demonstrated that V_B1_, V_C_ and V_A_ can decrease the binding affinity between Rv3291c(MtbLrpA) and DNA [31]. In addition, in our data we found that V_B1_ with a final concentration of 10 mM can inhibit the growth of BCG in vitro. By considering the importance of V_B1_, V_A_, V_C_ and V_D_, we proposed that lacking certain vitamins may play a role in bacterial persistence.

Therefore, in the present study, the transcriptomic and metabolomic analyses were conducted on *M. bovis* BCG after V_B1_ induction, by using RNA-sequencing and LC-MS platforms, respectively. The results indicated that V_B1_ exhibited an inhibitory effect on the growth of BCG in vitro. Candidate genes involved in the cell wall formation, energy metabolism and metabolic regulation were differentially expressed in BCG cultures in the absence and/or presence of V_B1_. Furthermore, the concentrations of amino acids and metabolites such as adenosine diphosphate (ADP), inosine and so on were decreased after V_B1_ treatment.

## Results

### Inhibitory effects of V_B1_ on BCG growth and its minimum inhibitory concentration

**Fig. 1:**
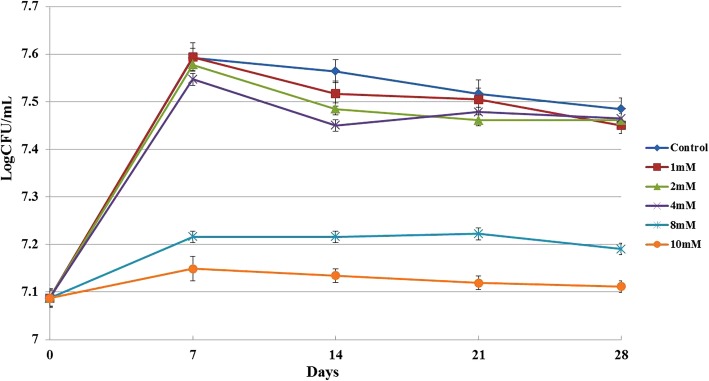
Effect of V_B1_ on BCG growth. BCG cultures were inoculated into 7H9 liquid medium, and treated with different concentrations of V_B1_ (1.0, 2.0, 4.0, 8.0, 10.0 mM). BCG cultures without V_B1_ treatment were used as controls. The OD_600nm_ value was detected every seven days. Each condition was tested in triplicates. X-axis represents the time (Unite is day), while Y-axis represents the log of CFU count

Previous study demonstrated that V_D_ can suppress the intracellular growth of *M. tuberculosis* in vitro [32]. In addition, the high dose of V_C_ was found to sterilize *M. tuberculosis* cultures in vitro [28]. In the present study, V_B1_ was added in certain medium at different final concentration of 1.0, 2.0, 4.0, 8.0, 10 mM, in order to test the effect of V_B1_ on BCG growth. It was observed that 8 mM V_B1_ has inhibited the growth rate of BCG (Fig. 1). In addition, the minimum inhibitory concentration (MIC) of V_B1_ was detected as 8 mM (data not shown).

### Genome expression analysis of BCG following V_B1_ treatment

**Fig. 2:**
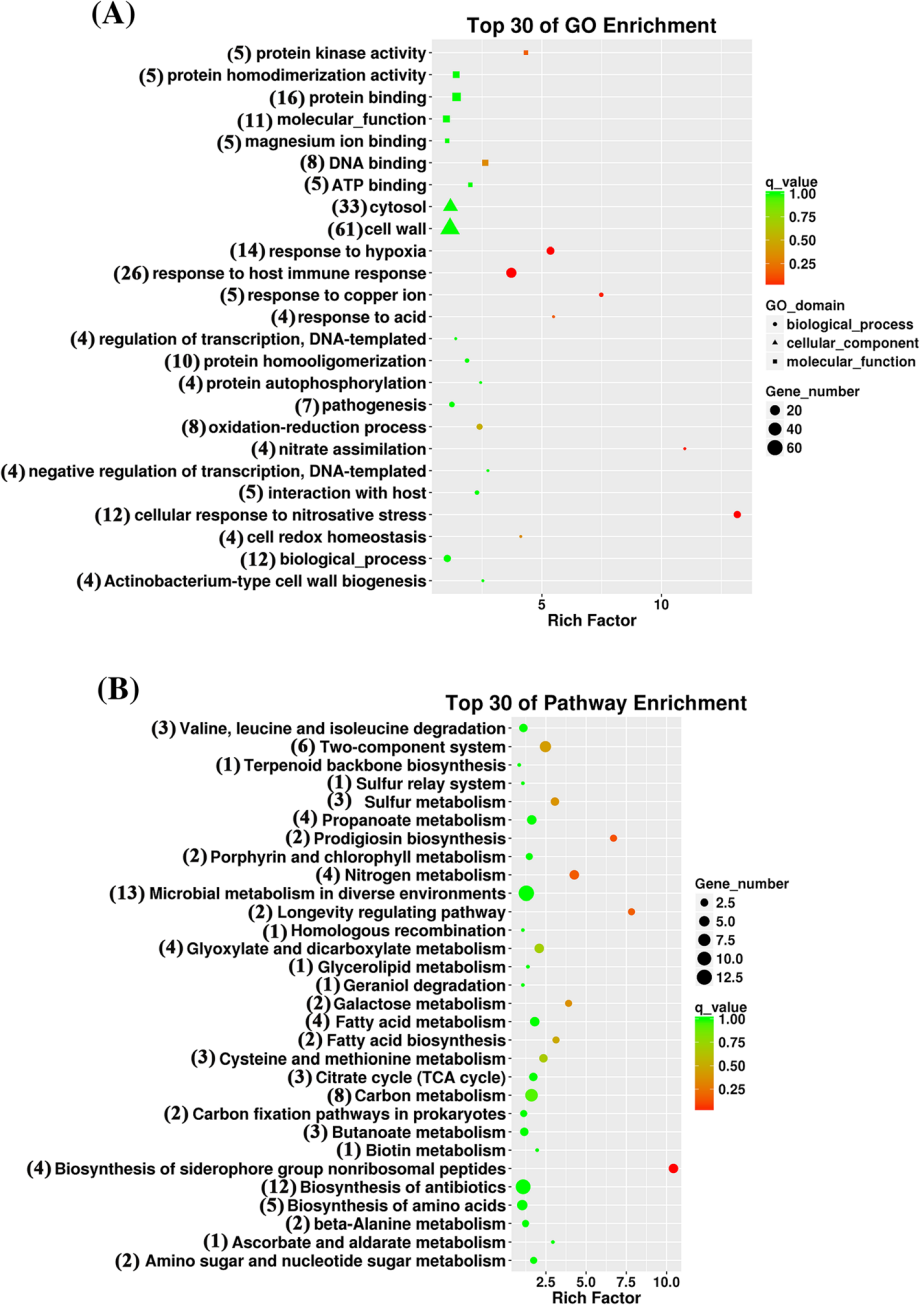
GO enrichment and KEGG pathway analysis of BCG treated with 8 mM V_B1_. (**a**) GO enrichment analysis. (**b**) KEGG pathway analysis. The numbers of genes in Top 30 of GO and Pathway Enrichment are listed on the left side of the figures, respectively

A total of 100 genes (2.5% of whole genome) were induced, whereas 160 genes (4% of whole genome) were repressed for more than 2-fold (Additional file 1: Table S3). Fold changes were calculated by the power of 2 for all the studied genes. For both GO and KEGG enrichment analyses, corrected P<0.05 was selected as a threshold to determine the significant enrichment of each gene set. The genes with an adjusted *P*-value of ≤0.05 were identified as differential expression. GO analysis of the transcripts was identified through Bio::SAGE::Comparison tool (https://metacpan.org/pod/Bio::SAGE::Comparison). The GO enrichment transcriptional analysis revealed that the differentially expressed genes in BCG strain treated with 8 mM of V_B1_ were significantly enriched in the following functional categories:“cell wall”, “cytosol”, “response to host immune response”, “cellular response to nitrosative stress”, “response to hypoxia”(Fig. 2a). On the other hand, KEGG analysis of the transcripts was performed by BLAST against KEGG database. KEGG enrichment analysis indicated that most of the variable genes were associated with the following biological processes: “microbial metabolism in diverse environments”, “biosynthesis of antibiotics”, “carbon metabolism”, “two–component system”, “biosynthesis of amino acids”, “nitrogen metabolism” and “fatty acid metabolism”**(**Fig. 2B). The major identified functional groups are discussed below.

### NarGHJI operon

The 4-gene *NarGHJI* operon, *JTY_RS06200* (*narG*) to *JTY_RS06215* (*narI*) were downregulated at different degree and *NarG* was repressed 2-fold in V_B1_ treated culture (Additional file 2: Table S7)**.** *NarGHJI* operon encodes for nitrate reductase, an enzyme that reduces nitrate to nitrite during the absence of oxygen, representing the first step of ammonification and denitrification [33]. Nitrogen and nitrogen compounds play an important role in the battle between hosts and pathogens. Nitrate reductase utilizes reactive nitrogen intermediates to promote the growth of Mtb in macrophages. During this process, NarG encoding nitrate reductase may play an essential role. Interestingly, NarG is a conserved protein in mycobacteria, and can be used as an ideal taxonomic marker for sorting different species in mycobacterium [33]. Besides, NarG mutant strains have been used to infect the severe combined immunodeficient mice, which leads to less extensive tissue damage compared wild type strains, indicating that nitrate respiration contributes significantly to the virulence of *M. bovis* BCG [34].

### Regulators

Mtb exhibits an increased pathogenicity due to its ability to adopt a dormant state, which may retain a lifelong risk of latent tuberculosis infection. The regulators play an important role in response to the surrounding environmental conditions, by changing the expression levels of the corresponding genes. In the present study, a total of 20 genes constituting approximately 10% of all BCG regulators were significantly altered in response to V_B1_ treatment. It is noticeable that five group of two-component transcription regulators were significantly changed in BCG cells after V_B1_ treatment.

The downregulation of *JTY_RS10535* (*Rv2034*) may regulate the expression of *dosR* gene and plays a significant role in lipid metabolism and hypoxic adaptation [35]. In addition, *Rv2034* is one of the in vivo expressed Mtb antigens (IVE-TB), which is considered as the potential TB vaccine candidate antigen. This observation is based on the expression of *Rv2034* during the process of inflammatory pulmonary infection and the specificity of cloned T cells for *Rv2034* to inhibit Mtb outgrowth from infected monocytes [36, 37]. The downregulated *JTY_RS18665* (*Rv3574*) is a member of TetR family transcriptional regulators, which is highly conserved in the mycobacteria. This multiple stress responsive transcriptional regulator controls a number of regulons that participate in the lipid degradation and induces the corresponding genes associated with lipid metabolism. Moreover, *JTY_RS18665* (*Rv3574*) positively regulates KstR and potentially acts as the main regulator of lipid metabolism during hypoxia-induced dormancy [38, 39].

In the Mtb transcriptional regulatory network, *JTY_RS00450* (*Rv0081*) represents an essential regulatory hub and modulates the response to hypoxia in the long-term survival of *M. tuberculosis* [40]*.* Previous study has reported that the incubation of this protein with whole blood isolated from latently infected individuals may stimulate the activation of IFN-γ, suggesting the antigenic properties of this protein [41]. In V_B1_-treated culture, *JTY_RS00450* (*Rv0081*) was downregulated by 13-fold (Additional file 2: Table S2).

DosR, a crucial regulator, belongs to two component regulatory system, which enhances dormancy adaptation of *M. tuberculosis* under low oxygen conditions and is responsible for latent tuberculosis infection. Upon exposure to hypoxia, nitric oxide, carbon monoxide, or ascorbic acid, DosR induces the expression of 48 genes that are referred as DosR regulon, including *dosRS*, *hspX*, *narK2*, *tgs1* and so on [42–46]. However, only a few of these regulon have been functionally characterized, while most of them are of unknown function [47]. A recent study has demonstrated the role of DosR regulon in metabolic adaptation, which is important for Mtb to shift from an actively respiring to dormancy [48]. Hence, the function of DosR regulon is indispensable for bacterial survival during the dormant state and *JTY_RS16225* (*Rv3133c*) was repressed by 5-fold in V_B1_-treated culture in this study (Additional file 2: Table S2).

When *M. tuberculosis* is in its dormant state, this pathogen can persist in a hostile environment through many different ways. One of these is the rapid downregulation of ribosome biogenesis to match the decreased translational demand, which requires the coordinate transcriptional regulation of the synthesis of all ribosome elements. CarD is an essential protein that modulates the mycobacterial rRNA transcription. Loss of CarD has been reported to be lethal for mycobacteria in vitro and during infection of mice. Moreover, CarD depletion may increase the susceptibility of *M. tuberculosis* to being killed by starvation, oxidative stress and DNA damage, accompanied by failure to reduce rRNA transcription [49]. In this study, *JTY_RS18710* (*Rv3583c*) was decreased more than 2-fold in V_B1_ supplemented culture (Additional file 2: Table S2).

The metabolic regulator *JTY_RS17900* (*WhiB3*) was up-regulated more than 2.4-fold in V_B1_-treated mycobacterial cells (Additional file 2: Table S2). WhiB3 includes the 4Fe-4S (iron-sulfur) cluster proteins and serves as a redox sensor and effector molecule, which regulates the redox homeostasis and virulence via interaction with specific host gases (e.g. O_2_ and NO) [50]. The expression of *WhiB3* is drastically increased upon acid stress, while weakly induced upon exposure to oxidants during the growth of Mtb under different environmental pressure conditions [51]. In V_C_ and H_2_O_2_-treated mycobacterial cells, the expression of *WhiB3* is induced more than 2-fold. Thus, both V_C_ and peroxide can stimulate the expression of the corresponding protein [4, 52, 53].

Under nitric oxide treatment and macrophage infection, the expression level of transcription regulator *JTY_RS20155* (*WhiB6*) is highly upregulated [54]. WhiB6 is able to regulate the expression of ESX-1 during mycobacterial dissemination and granuloma formation in zebrafish embryo model [55]. Through comparative transcriptomic analysis, *WhiB6* has been shown to regulate aerobic and anaerobic metabolism, virulence and cell division [54]. In V_B1_-treated cells, *JTY_RS20155 (Rv3862c)* was upregulated more than 3-fold (Additional file 2: Table S2).

The repressor *JTY_RS20120 (Rv3855)* belonging to TetR/CamR family transcriptional regulators was upregulated by approximately 2-fold in V_B1_-treated culture (Additional file 2: Table S2). In *Mycobacterium* genome, *Rv3854c* (*ethA*) and *Rv3855* (*ethR*) can confer to the resistance to antitubercular drug ethionamide (ETH) among patients infected with MDR strains of *M. tuberculosis*. In addition, the ethA-ethR-deficient *M. bovis* BCG mutant displays an increased adherence to mammalian cells and enhanced persistence in vivo [56]*.*

*Rv3066* is one of TetR family regulator and represses the transcription of *mmr* by directly binding to the inverted repeat (IR) of promoter [57], *Mmr (Rv3065)* is a multidrug efflux pump, which belongs to the SMR (small multidrug resistance) family. Mmr can modulate the resistance to some toxic compounds, including ethidium bromide, acriflavine, safranin O, pyronin Y, tetraphenylphosphonium and thioridazine [58, 59]. Furthermore, Mmr is able to recognize and eliminate a large range of antimicrobial agents, thus leading to the intrinsic resistance to antimicrobials in Mtb. The results of transcriptomics analysis demonstrated that *JTY_RS15890* (*Rv3066*) was upregulated by 2-fold in V_B1_-treated cells (Additional file 2: Table S2).

### DNA replication, translation and repair

The *M. tuberculosis* genome encodes an NAD^+^-dependent DNA ligase (*ligA*) and three diverse ATP-dependent ligase homologs, namely, *ligB*, *ligC*, and *ligD* [60]*.* Two of them are repressed in V_B1_-treated BCG cultured cells, suggesting that the reduced growth rate of BCG was possibly due to the retardation of replication process.

The detected genes were also involved in the process of translation, such as those encoded by 50S ribosomal proteins (i.e. *rpmB1*, *rplE*, *rplD*, *rplC, rplW and rplB*) were downregulated, leading to a decrease in the demand for mRNA and protein synthesis during the state of dormancy. In addition, *JTY_RS00440* (*Rv0079*), a member of DosR regulon, is involved in the regulation of translation initiation via the interaction between bacterial ribosomal subunits and its products in order to inhibit the protein synthesis and bacteria growth [61–63]. It was observed that the expression level of *Rv0079* was 6-fold lower after V_B1_ treatment (Additional file 2: Table S2).

In addition, high-level expression of chaperone proteins under various stress conditions may prevent the irreversible loss of protein function [64]. GroEL is believed to function as chaperonin cage, which can alter the rate of protein folding [65]. Although the mechanism underlying GroELs-facilitated protein folding is not clearly understood, it has been observed that these GroELs do not demand ATP to promote protein folding [66]. In this study, *JTY_RS02300* (*Rv0440*) that encodes molecular chaperone GroEL was identified to be upregulated 2-fold in V_B1_-treated samples (Additional file 2: Table S2).

Response to DNA damage is crucial for bacterial survival. The *JTY_RS14065* (*Rv2720*) *LexA* is a key element of SOS regulatory network involved in the bacterial response to DNA damage, which is commonly found among bacteria and displayed a substantial discrepancy in transcriptional regulation [67]. In V_B1_-treated BCG culture, the expression level of *lexA* was decreased, suggesting that the activation of DNA repair system in BCG is essential for dormancy survival.

### Energy metabolism (fatty acid/cholesterol/glycolipid)

Acquisition and utilization of nutrients within the host cells is one of the main factors contributing to the long-term persistence of Mtb. Certain lipid precursors, such as cholesterol are required for the host cells, which can promote the survival of Mtb and its infection [68]. The results of transcriptomic analysis indicated that the majority of the genes related to cholesterol metabolism were downregulated in V_B1_-treated BCG culture. For example, *JTY_RS18520* (*fadA5*) encoding for acetyl-CoA-acetyltransferase is involved in the catabolism of cholesterol, which provides a carbon source for energy production. In the infectious mouse model, the *fadA5* mutant showed an attenuated disease phenotype as compared to the wild-type strain during chronic infection. Therefore, *fadA5* is considered crucial to the survival of Mtb in vivo, and can be used as a potential drug target [69]. Additionally, *JTY_RS18515* (*cyp125*), a cholesterol hydroxylase gene, is encoding cytochrome-P450-steroid-C27-monooxygenase, which involved in the cholesterol catabolism of *M. bovis* BCG, Mtb and other strains [70].

*JTY_RS18475* (*kstD*) encodes 3-oxosteroid-1-dehydrogenase that regulates cholesterol through 4-androstene-3,17-dione/1,4-androstadiene-3,17-dione (AD/ADD) pathway. Deletion of *kstD* gene may suppress Mtb growth due to the inactivation of cholesterol degradation pathway.

*JTY_RS16210* (*tg1*) encodes diacylglycerol-O-acyltransferase enzyme and is potentially encoding triacylglycerol synthase (TGS). Triacylglycerol (TG) is widely used as an energy source for the intracellular growth of pathogen. It is reported that the accumulation of TG is essential for the metabolism of Mtb [71]. In this study, the expression levels of *Rv3130c* (*tg1*) were downregulated 14-fold in BCG culture following V_B1_ treatment (Additional file 2: Table S2).

Nitrogen and nitrogen compounds play an important role in the battle between *M.tuberculosis* and host cells. *M.tuberculosis* can utilize nitrate as a final electron acceptor under anaerobic conditions to enhance its survival. Certain genes involved in nitrogen assimilation such as *JTY_RS01345* (*Rv0253*), *JTY_RS06200* (*Rv1161*) and *JTY_RS01340* (*Rv0252*) were repressed more than 2-fold in V_B1_-treated cells (Additional file 2: Table S2). Besides, the expression levels of operon *NarGHJI* encoding nitrate reductase were also decreased.

Through metabolic adaptation, *M. tuberculosis* encounters a range of different microenvironments in its host. Glucose is one of the most abundant and essential carbon sources for many pathogenic bacteria such as *M. tuberculosis*. In *M. tuberculosis*, glycolytic pathway is highly conserved and fructose-6-phosphate phosphorylation is the key step in glycolysis process catalyzed by phosphofructokinase (PFK) activity. The genomic analysis predicted that there are two genes, namely, *pfkA (rv3010c)* and *pfkB (rv2029c)*, encoded for putative PFK. Recent studies demonstrated that PFKA of Mtb accounted for the overall PFK activity without functional redundancy of PFKB. Notably, the minor isoenzyme PFKB of *E. coli* is responsible for around 10% of PFK activity. It is thus possible that Mtb PFKB possesses the ability to phosphorylate sugar-based substrates in addition to fructose-6-phosphate [72]. In V_B1_ treated culture, *JTY_RS10510* (*rv2029c*) was downregulated by approximately 50-fold (Additional file 2: Table S2).

In overall, the majority of these genes are participated in energy metabolism, including the fatty acid, cholesterol and glycolipid metabolism. It is postulated that pathogens downregulated its metabolic activity to reduce energy consumption and to persist in a prolonged dormant state.

### Ion-related protein

The balance of metal ions (e.g. iron, copper, zinc and magnesium) concentrations in the host cell is crucial for the adaptation to the environmental changes during the host responses to pathogen invasion. Bacterial growth is involved in a series of biological actions in terms of storage, transport and the use of metal ions. Recently, copper has been proposed to be used directly as a host defense mechanism, since a high concentration of copper was found in the mycobacterial phagosome [73]. The elevated copper levels are toxic and harmful to the cells through the induction of oxidative stress via Fenton reactions [74]. In addition, high copper levels can induce Fe-S clusters destabilization [75] and replace metal cofactors in proteins. Nevertheless, copper has been shown to act as a metalloenzyme cofactor and is mainly involved in controlling all types of cellular activities [76].

*JTY_RS05125* (*csoR*) is an essential gene that is necessary for the retention of copper homeostasis in Mtb, while the target deletion of *csoR* is able to promote survival of Mtb during the early stages of chronic infection [77]. However, *csoR* has been shown to repress the expression of *cso* operon in the absence of copper [78]. In the present study, *cso* operon (*csoR-Rv0968-ctpV-Rv0970*) was up-regulated by approximately 2-fold in V_B1_-treated BCG cultured cells (Additional file 2: Table S2**)**. Moreover, *ctpV* (*JTY_RS05135*) potentially functions as a copper exporter and is essential for copper detoxification in Mtb. In animal models, *ctpV* deletion mutant exerts a potent strong effect on host immune response in Mtb [79]. Overall, these findings suggest that *M. bovis* BCG is able to adapt to V_B1_ treatment conditions, by regulating the copper concentrations, control the exports of copper and retain a safe intracellular environment for Mtb to maintain its dormant phase.

### Transport

ABC transporters are crucial for survival of Mtb, with regard to nutrients uptake and toxicants efflux. In Mtb genome, approximately 2.5% genes encode the ABC transporter proteins, including peptide transporters, macrolide transporters, amino acid transporters, carbohydrate transporters, iron transporters, anion transporters and so on [80]. In particular, there are two peptide permease operons *dpp* (*rv366c-rv3663c*) and *opp* (*rv1283c-rv1280c*) in the genomic sequence of *M. tuberculosis* H37Rv [81]. Both contain two nucleotide-binding subunits and integral membrane proteins, and one substrate-binding polypeptide [82]. The *rv3665c-rv3662c* encodes for the oligopeptide transporter of *M. tuberculosis,* basing on the fact that an *rv3665c-rv3662c* knockout mutant is resistant to bialaphos [83]. Microarray expression profiling has clearly indicated that the *rv3665c-rv3663c* locus regulates at least some genes which are induced during nutrient deprivation and hypoxia [81]. In the present study, our expression results indicated that *dppA-dppB-dppC-dppD* (*Rv3666c-Rv3665c-Rv3664c-Rv3663c*) genes were all downregulated.

Mtb UspABC transporter, one of four Mtb ABC transporters, has been shown to be essential for the growth of *M. tuberculosis* in vitro [84]*.* UspABC transporter may have a key function in the assimilation of amino sugars that enables Mtb to optimize the use of limiting nutrients during intracellular infection [85]. This observation supports the possible role of UspABC transporter in the recycling ingredient of cell-wall peptidoglycan [85]. Furthermore, our data demonstrated that the expression levels of *uspABC* operon were downregulated more than 2-fold in BCG culture supplemented with V_B1_ (Additional file 2: Table S2).

### Cell wall formation

The cell walls of Mtb have a high lipid content, which are composed predominantly of mycolic acids. Cell walls may play an important role in Mtb pathogenesis through the control of its own cellular processes, including neutralization of free radicals, alterations in membrane permeability and modulation of host immune response [86]. Mycolic acids are able to increase host survival rates and reverse antibiotic resistance, due to low permeability of cell walls. Therefore, identification of the major enzymes involved in mycolic acid biosynthesis may lead to the development of novel drug targets for TB treatment. In Mtb, there are six *accD* genes encoding for acyl-CoA carboxylases. Notably, *JTY_RS05160* (*rv0974c*) or known as *accD2*, is believed to take part in mycolic aicd biosynthesis. Cell wall-bound mycolic acids were absent in *accD2* and *accD3* mutants, suggesting that these mutants are indeed deficient for mycolic acid synthesis [87]. The transcriptomic analysis demonstrated that *rv0974c* was almost 4-fold lower when treated with V_B1_ (Additional file 2: Table S2).

*JTY_RS10525* (*Rv2032c*) or *acg* gene, encoding the NAD(P) H nitroreductase, was downregulated about 30-fold in V_B1_ treated BCG culture (Additional file 2: Table S2). Deletion of *acg* produced a mutant that is attenuated in both macrophage and murine model infections. However, *acg* deletion did not come to terms with the viability of the mutant in vitro and in vivo, under oxidative and nitrosative stresses. Moreover, the *acg* mutants are more sensitive to antibiotic drugs (e.g. nitrofurantoin and nitrofuran) than the WT strain. Thereby, *acg* is a key virulence factor for Mtb, which enhances the growth and survival of Mtb in macrophages and mice [88].

### PE and PPE families proteins

PE and PPE are glycine-rich proteins that constitute approximately 10% of Mtb proteome. However, the function of these glycine-rich proteins is unknown [89]. Recent studies have proposed that these two protein families may contribute to the bacterial persistence in granulomas, the bacterial survival in macrophages and the antigenic change [90, 91]. During the infection process, phagocytes can kill intracellular microorganisms via the inhibition of phagosome acidification. Further, this prevents the fusion between lysosome and phagosome [92]. It has been reported that a strain lacking *JTY_RS09290* (*Rv1787*) is attenuated in vivo and does not inhibit the phagosome-lysosome fusion and the acidification of vacuole, suggesting that this protein may be involved in the development of bacterial intracellular environment and the formation of phagosome [93]. In V_B1_-treated culture, the expression levels of *Rv1787* were upregulated by approximately 2-fold (Additional file 2: Table S2).

In addition, it has been reported that *JTY_RS1794*5 (*Rv3425*) is encoded by an open reading frame located in the RD11 region of Mtb genome and this protein is expressed during the exponential growth in vitro [94]*. Rv3425* exhibited both IgG1 antibody response and Th1/Th2 immune response following immunization in mice, supporting that this protein is involved in the production of a potent cellular and humoral immune response. Moreover, vaccines containing Ag85B and Rv3425 antigens promote the effects of immunity and confer a protection against Mtb infection in C57BL/6 mice [95]. Therefore, Rv3425 may be a promising vaccine candidate for TB treatment. In the present study, V_B1_ treatment induced a 3-fold increase in Rv3425 expression levels in BCG culture as compared to the control sample (Additional file 2: Table S2).

*JTY_RS10910* (*Rv2108*) can be used for the rapid detection and identification of mycobacteria complex containing *M. africanum*, *M. bovis* and *M. bovis BCG* strain, due to its high specificity towards Mtb complex [96]. In a previous study, the recombinant p27-PPE36 (Rv2108) protein is immunologically active and reacts with antibodies in the sera of TB patients, as detected by ELISA assay [97]. It is worthwhile to note that the presence of IgA and absence of IgG antibody responses can be used as the specific diagnosis markers for Mtb [98]. The current transcriptomic data indicated that the expression levels of PPE36 were upregulated more than 3-fold in V_B1_-treated cells (Additional file 2: Table S2).

### Hypothetical protein

The transcriptomic analysis indicated that the expression levels of the 260 genes were altered in the bacterial cells following V_B1_ treatment. Among these genes, 51 (19%) were encoded for hypothetical proteins. The functions of several hypothetical proteins have been reported, but most of them are of unknown function. For example, the conserved hypothetical genes *JTY_RS09425* (*Rv1813c*) and the co-transcribed *JTY_RS09420* (*Rv1812c*) are encoded for dehydrogenase. *Rv1813c* is predicted to be related with Mtb virulence in vivo [99]. In this study, both genes were downregulated at a factor of higher than 9-fold and 2-fold, respectively. Moreover, *JTY_RS10515 (Rv2030c)*, *JTY_RS16955 (Rv3269)*, *JTY_RS00440 (Rv0079c)*, *JTY_RS09435 (Rv1815)* and *JTY_RS13660* (*Rv2627c*) were downregulated more than 3-fold. In contrast to those genes, *JTY_RS12370* (*Rv2390c*), *JTY_RS00960* (*Rv0179c*), *JTY_RS08085* (*Rv1535*), *JTY_RS13125* (*Rv2525c*) and *JTY_RS09350* (*Rv1799*) were upregulated more than 2-fold (Additional file 2: Table S2).

### Universal stress protein

There are 10 universal stress proteins (ups) in *M. tuberculosis*, most of the function are unknown. Recently, proteomic and transcriptomic analyses have revealed that a number of *usp* genes are significantly upregulated under nitric oxide, carbon monoxide and hypoxia conditions, as well as during *M. tuberculosis* infection of macrophage cell lines [100]. It is worth to mention that the expression levels of 10 universal stress proteins such as *JTY_RS10505* (*Rv2028c*), *JTY_RS16230* (*Rv3134c*), *JTY_RS13645* (*Rv2624c*), *JTY_RS13640* (*Rv2623c*), *JTY_RS10325* (*Rv1996*), *JTY_RS10375* (*Rv2005c*), *JTY_RS12040* (*Rv2319c*), *JTY_RS08515* (*Rv1636c*), *JTY_RS10495* (*Rv2026c*) and *JTY_RS05465 (Rv1028c*) were repressed at different magnitudes. Among these stress proteins, *Rv2028c*, *Rv1996* and *Rv2005c* that belong to *M. tuberculosis* DosR regulon were repressed at 36, 7 and 2 folds, respectively, among the V_B1_-treated BCG culture (Additional file 2: Table S2).

### RT-qPCR verification for the expression of selected genes

**Fig. 3:**
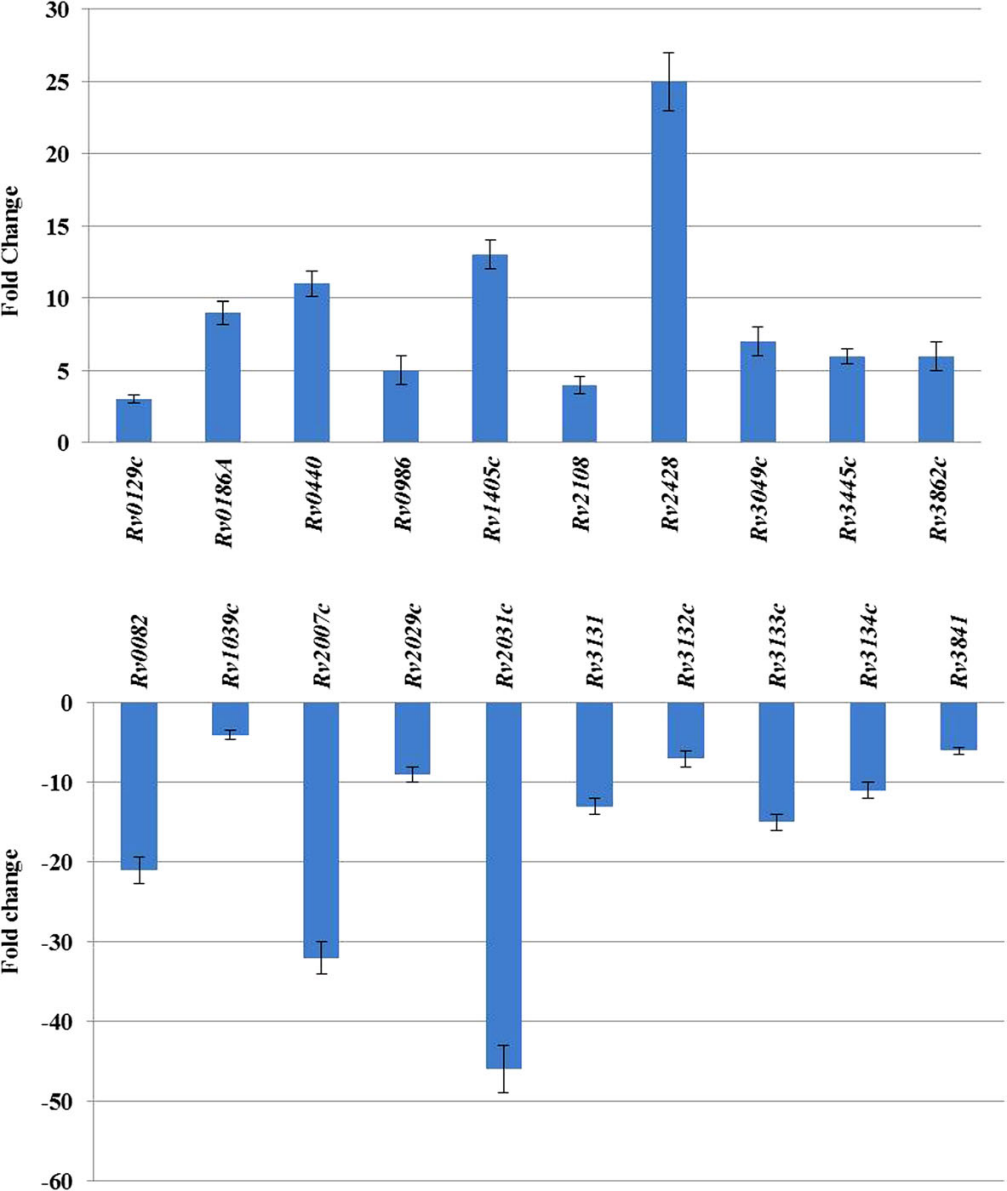
The relative expression of selected genes. The relative expression levels of selected genes in the presence and absence of 8 mM V_B1_ by qRT-PCR analysis. Standard deviations (calculated for three biological replicates) are indicated. X-axis represents the identities of tested genes; Y-axis represents the fold-changes of tested genes in both V_B1_ treatment and control groups. **a** The fold-changes of upregulated genes following V_B1_ treatment. The genes listed on the X-axis are *JTY_RS00695* (*Rv0129c*), *JTY_RS01000* (*Rv0186A*), *JTY_RS02300* (*Rv0440*), *JTY_RS05235* (*Rv0986*), *JTY_RS07460* (*Rv1405c*), *JTY_RS10910* (*Rv2108*), *JTY_RS12600* (*Rv2428*), *JTY_RS15805* (*Rv3049c*), *JTY_RS18025* (*Rv3445c*) and *JTY_RS20155* (*Rv3862c*). **b** The fold-changes of downregulated genes following V_B1_ treatment. The genes listed on the X-axis are *JTY_RS00455* (*Rv0082*), *JTY_RS05530* (*Rv1039c*), *JTY_RS10385* (*Rv2007c*), *JTY_RS10510* (*Rv2029c*), *JTY_RS10520* (*Rv2031c*), *JTY_RS16215* (*Rv3131*), *JTY_RS16220* (*Rv3132c*), *JTY_RS16225* (*Rv3133c*), *JTY_RS16230* (*Rv3134c*) and *JTY_RS20045* (*Rv3841*)

A total of 20 genes were selected for qRT-PCR examination in order to confirm the obtained transcriptomic data (Fig. 3). The primers used in the qRT-PCR assay are listed as the supplementary materials (Additional file 3: Table S1). The alteration in the levels of expression corresponded to the results of sequencing.

As illustrated in Fig. 3, the genes *JTY_RS10385* (*Rv2007c*), *JTY_RS10520* (*Rv2031c*), *JTY_RS16215* (*Rv3131*), *JTY_RS16230* (*Rv3134c*), *JTY_RS16225* (*Rv3133c*), *JTY_RS05530* (*Rv1039c*), *JTY_RS20045* (*Rv3841)*, *JTY_RS16220* (*Rv3132c*), *JTY_RS00455* (*Rv0082*), *JTY_RS10510* (*Rv2029c*) encoding ferredoxin, alpha-crystallin, NAD(P) H nitroreductase, universal stress protein, transcriptional regulator, PPE family protein, ferritin BfrB, histidine kinase, oxidoreductase and phosphofructokinase were downregulated at 32, 46, 13, 11, 15, 4, 6, 7, 21, 9 folds, respectively. On the contrary, the genes *JTY_RS12600* (*Rv2428*), *JTY_RS07460* (*Rv1405c*), *JTY_RS15805* (*Rv3049c*), *JTY_RS20155* (*Rv3862c*), *JTY_RS02300* (*Rv0440*), *JTY_RS10910* (*Rv2108*), *JTY_RS01000* (*Rv0186A*), *JTY_RS05235* (*Rv0986*), *JTY_RS18025* (*Rv3445c*), *JTY_RS00695* (*Rv0129c*) encoding alkyl hydroperoxide reductase subunit C, methyltransferase, NAD(P)/FAD-dependent oxidoreductase, transcriptional regulator, molecular chaperone GroEL, PPE family protein, metallothionein, ABC transporter ATP-binding protein, ESAT-6 like protein EsxU and diacylglycerol acyltransferase were upregulated at 25, 13, 7, 6, 11, 4, 9, 5, 6 and 3 folds, respectively **(**Fig. 3).

### LC-MS analysis of amino acids and metabolites

By considering that most of the amino acid metabolic pathways were altered, we aimed to investigate the effects of individual amino acid levels in the V_B1_-treated and wild strain BCG cultures. The results of LC-MS demonstrated that the concentrations of most amino acids, except for methionine, were higher than 1.0-fold in the V_B1_-treated group as compared to the untreated group (Additional file 4: Table S4).

**Fig. 4:**
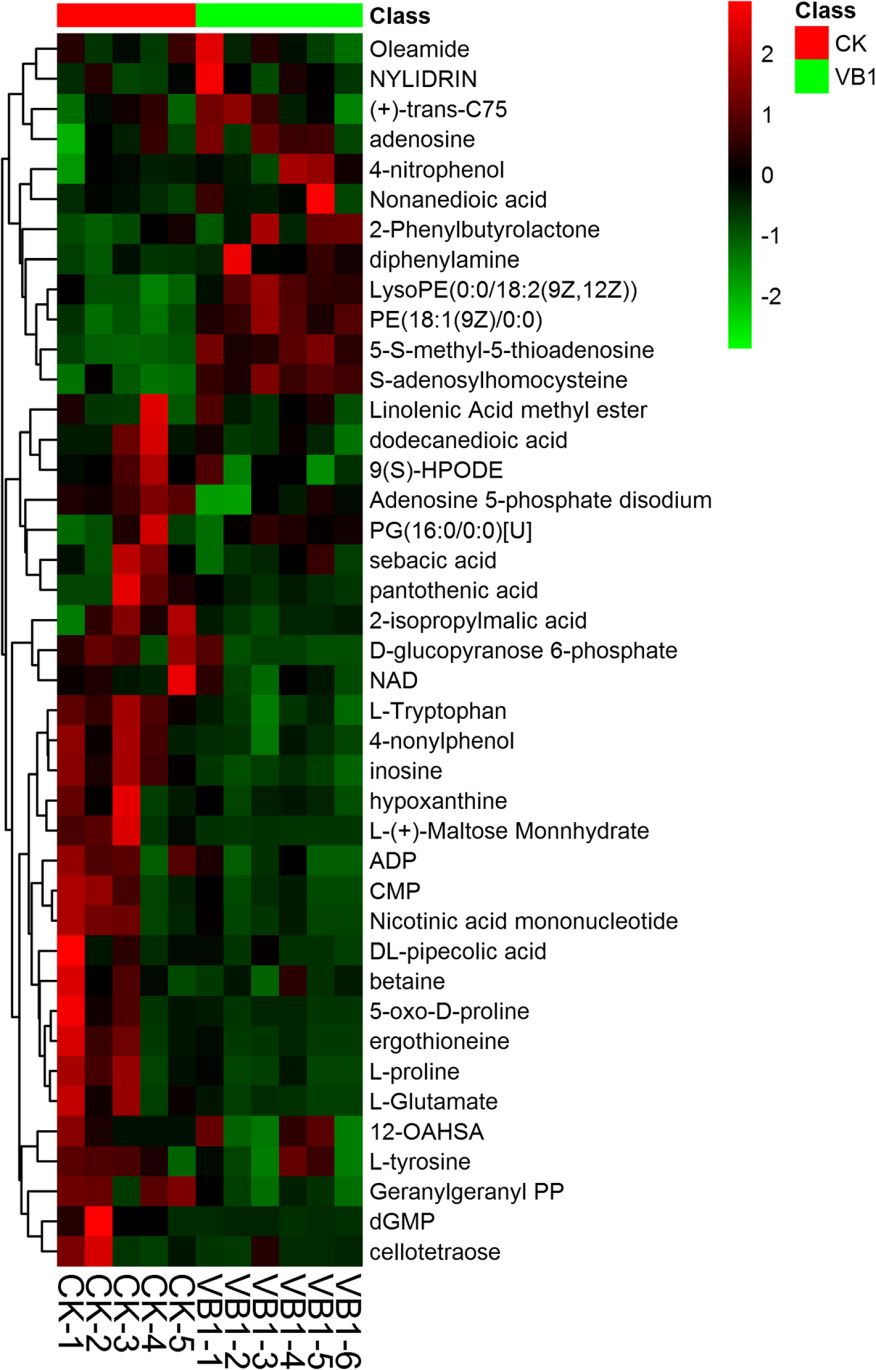
The heatmap analysis of different metabolites in V_B1_ treatment and control groups. The red and blue colors indicate metabolites with increased and decreased levels, respectively. The rows represent control samples (CK-1, CK-2, CK-3, CK-4 and CK-5) and experimental samples treated with V_B1_ (V_B1_–1, V_B1_–2, V_B1_–3, V_B1_–4, V_B1_–5 and V_B1_–6). The identities of metabolites are listed on the right side of the heatmap

In addition to amino acids, the metabolites were also detected by LC-MS. Each condition has 6 samples except for 1 sample in control group was ommited because the problem of samples. The heatmap analysis was shown in Fig. 4. Of these, four metabolites, namely, 5-S-methyl-5-thioadenosine, PE (18:1(9Z)/0:0), S-adenosylhomocysteine and LysoPE (0:0/18:2(9Z,12Z)) were upregulated at least 2-fold, whereas the remaining metabolites including L-tryptophan, inosine, geranylgeranyl PP, adenosine 5-phosphate disodium, 4-nonylphenol and ADP were downregulated by 2-fold (Additional file 5: Table S5).

### The associated KEGG pathway for both transcriptomics and metabolomics data

For the analysis of both transcriptomics and metabolomics data, we explored the KEGG pathway involved in the primary and secondary metabolism of *M. bovis* BCG after V_B1_ induction. Differentially expressed genes and metabolites mainly participated in 9 KEGG pathways, including nitrogen metabolism, purine metabolism, methane metabolism, cysteine and methionine metabolism, secondary metabolites biosynthesis, terpenoid backbone biosynthesis, oxidative phosphorylation, photosynthesis and metabolic pathways. The metabolite compounds such as S-adenosylhomocysteine, geranylgeranyl PP, inosine and ADP fluctuated in BCG culture after treated with V_B1_. Moreover, the number of expressed genes involved in metabolic pathways, biosynthesis of secondary metabolites, nitrogen metabolism, purine metabolism, methane metabolism, oxidative phosphorylation and terpenoid backbone biosynthesis were 572, 295, 21, 60, 24, 43 and 27, respectively. All the corresponding genes were downregulated, and the concentrations of ADP, inosine and geranylgeranyl PP were also decreased (Additional file 6: Table S6).

Nitrogen is a basic biomolecule for amino acids, nucleotides, cell wall components and cofactors. Nitrogen metabolism plays an essential role in the life cycle of *M. tuberculosis*. In this study, the 21 genes participating in nitrogen metabolism were downregulated and the concentration of ADP was decreased in V_B1_-treated culture.

Despite their fundamental importance in forming the building blocks for DNA and RNA, purine metabolites may supply the cells with energy and cofactors to promote cell survival and multiplication [101]. The 60 genes involving in purine metabolism were downregulated at different magnitudes in the V_B1_-treated culture. Similarly, the concentrations of ADP and inosine metabolites were reduced.

## Discussion

Vitamin B consists of eight well-recognized members with high water solubility, which is involved in various metabolic pathways and host immunization. V_B1_ is a pivotal cofactor for all living microorganisms and exerts a significant function in the activation of immunization. The administration of V_B1_ as a dietary supplement can reduce the incidence of many types of cancer [102]. In addition, V_B1_ can be used to treat Parkinson’s disease via multiple mechanisms of action, including oxidative stress, inflammation, protein expression and cellular metabolism. The therapeutic applications of V_B1_ have extended to the prevention of immune HIV-associated complications. V_B1_ exerts beneficial effects in HIV patients through the protein expression of vascular endothelial growth factor and matrix metalloproteinase [103, 104]. In the present study, the physiological outcome of V_B1_ treatment in *M. bovis* BCG was examined by both transcriptomic and metabolomic analyses. The findings indicated that V_B1_ treatment could alter various cellular processes involved in the bacterial cell growth. Firstly, V_B1_ reduces lipid degradation and results in low energy supply, via the downregulation of genes involved in energy metabolism. Secondly, the genes related to cell wall formation, cell division and replication were downregulated, supporting that V_B1_ treatment reduces the growth of BCG. Thirdly, the expression levels of the genes encoding ribosomal synthesis were decreased in BCG cells treated with V_B1_. These results demonstrated that V_B1_ treatment may influence the protein synthesis apparatus and reduce protein synthesis, leading to slow bacterial growth. Furthermore, the expression of regulators modulating various cellular processes (e.g. lipid metabolism, lipid degradation, transport and stress response) were altered in BCG culture following V_B1_ treatment. These data suggests that bacteria can adapt to environmental stimuli via many different regulators by using distinct mechanisms of growth inhibition. Lastly, it is important to note that *NarGHJI* operon were repressed in BCG treated with V_B1_, indicating that the process of nitrogen assimilation considerably slows down as a consequence of reduced energy sources.

LC-MS based metabolomic analysis revealed that the concentrations of amino acids (AA), except for methionine, were lower in the V_B1_-treated culture than in the untreated culture. In fact, AA are used to support the physiological functions of pathogen, and the variation in AA availability significantly affects the expression of virulence factor and the growth of pathogen [105]. For instance, aspartate is a primary nitrogen source that enhances the virulence of *M. tuberculosis* during colonization of its host [106, 107]. Asparagine is another important nitrogen source for pathogen, which can be captured and digested by AnsP2 and AnsA. Digestion of asparagine may cause pathogens to resist the host immune defense and improve their survival rates in the host [108]. Moreover, alanine can be broken down to pyruvate and ammonium by alanine dehydrogenase, and has been used as an unique nitrogen source for the growth of *M. tuberculosis*, *M. avium* and *M. smegmatis* [109]. Both alanine and serine can prevent the growth of BCG strains in vitro by inhibiting glutamine synthetase activity. This enzyme plays an important role in bacterial nitrogen metabolism, metabolizing nitrogen through aminate glutamate in an ATP-dependent reaction [110]. Furthermore, when glycine, asparagine and aspartate are used as the nitrogen source, *M. bovis* BCG can grow rapidly in Sauton medium [111]. More importantly, AA acts as the basic substrate for the synthesis of protein and other regulatory molecules such as nitric oxide, polyamines, and creatin. Besides, AA can regulate MAPK signaling pathways in controlling energy metabolism [112]. Recent studies have shown that AA plays an important role in the coordination of host innate immunity. For example, leucine and glutaminere are crucial in mediating T cell functions, including the activation and differentiation of Th1 and Th17 cells [113–115].

The combination of transcriptomics and metabolomics data indicated that a large number of annotated genes (*n* = 572) were involved in metabolic pathways, and the corresponding metabolites were identified as ADP, S-adenosylhomocysteine, inosine and geranylgeranyl PP. Next, a total of 295 genes were associated with secondary metabolite biosynthetic pathways, linking to the reduced metabolite levels of ADP and geranylgeranyl PP. Despite the aforementioned pathways, the genes and metabolites involved in nitrogen and purine metabolisms were downregulated. Notably, ATP which is essential for energy production and cofactors function was found to be downregulated by approximately 3.5-fold. Besides, purine and pyrimidine nucleotides are used to create the DNA and RNA. Purine nucleotides such as ATP is vital for cellular energy supply, while guanosine triphosphate (GTP) and cyclic AMP (cAMP) are the essential signaling molecules [116]. Additionally, purines can be integrated into more complex biomolecules and act as cofactors such as nicotinamide adenine dinucleotide (NAD) and coenzyme A [101]. In this study, inosine was downregulated at least 2-fold in V_B1_-treated BCG culture. Inosine is made up of deaminizing adenosine and can be released extracellularly under inflammatory conditions [117, 118]. Inosine has been reported to exert nutritional and neuroprotective functions in nerve cells, and stimulates mast cell degranulation by activating adenosine A3 receptor [119]. Moreover, inosine exhibits anti-inflammatory activity by inhibiting the release of inflammatory cytokine from activated T cells and promoting the formation of IL-10. Clinical trials have proposed that inosine may be a promising drug target for patients suffering from multiple sclerosis [120].

All in all, the changement of metabolites following V_B1_ induction suggests a downregulation of gene expression and metabolic pathways in energy metabolism. In addition, abnormal expression of nitrogen metabolism genes and lower concentration of ADP could possibly lead to the slower growth rate of BCG after being treated with V_B1_.

## Conclusions

Overall, the findings of this study revealed that V_B1_ treatment can inhibit the growth of *M. bovis* BCG, by modulating the cellular processes such as replication, transcription, translation, transport and energy metabolism. More importantly, the expression levels of certain antigens were altered in BCG cells following V_B1_ treatment. These results suggest that V_B1_ can affect BCG growth by targeting its cellular processes and confer a protection against Tuberculosis by promoting host immunity and stimulating antigen production. Furthermore, metabolomic analysis indicated that most of the amino acid biosynthetic pathways were altered. To the best of our knowledge, the present study is the first to investigate the effects of V_B1_ *M. bovis* BCG, and provides the molecular and metabolic bases to further understand the impacts of V_B1_ on *M.bovis BCG.*

## Methods

### Strains, culture conditions and MIC (minimum inhibitory concentration) of V_B1_

*Mycobacterium bovis* BCG str. Tokyo 172 strain was grown in Middlebrook 7H9 medium Becton Dickinson supplemented with 0.05% Tween-80 (V/V), 10% ADC (Albumin-Dextrose-Catalase, BD) and 0.2% (V/V) glycerol. When OD_600nm_ value reached exponential phase, V_B1_ with final concentration of 1.0, 2.0, 4.0, 8.0, 10 mM were added and incubated at 37 °C for 4 weeks under continuous shaking (100 rpm). At days 7, 14, 21 and 28, 100 μL of fresh BCG was spread on the appropriate plates, and then incubated at 37 °C for 4 weeks. After incubation, the number of colonies was count to determine the effect of V_B1_ on BCG growth. The experiments were performed in triplicate. For transcriptomics and LC-MS analysis, when OD600nm value reached 0.3, V_B1_ at the final concentration of 8.0 mM for the test group were added and incubated at 37 °C for 48 h. There are 3 and 6 biological samples respectively in transcriptomics and LC-MS experiments. The procedure of MIC determination is as following: BCG was cultured at 37 °C for about 10 to 15 days until an optical density (OD) of an 0.5 McFarland standard (1 × 10^7^ CFU/mL) reached. Then the suspension was diluted 100 fold to obtain a standard inoculum with 10^5^ CFU/mL. 100 μL of standard inoculum was inoculated onto 7H10 plates containing V_B1_ with final concentration of 0, 0.125, 0.25, 0.5, 1.0, 2.0, 4.0, 8.0, 10.0 mM at 37 °C for 21 days. Then the colony number were counted and the MIC value was confirmed.

### RNA extraction and purification

*M. bovis* BCG was cultured with 7H9 liquid medium in the presence and/or absence of 8 mM V_B1_. At time point (48 h), bacterial cells (control and V_B1_-treated) were collected and centrifuged. RNA isolation was carried out as described previously [31]. In brief, BCG pellets were lysed and homogenized by high-speed agitation in a bead mill with the presence of glass beads and lysis buffer to lyse the cells and release the RNA. Then total RNA was extracted using RNeasy Mini Kit (Qiagen) following the instructions provided by the manufacturer with one on-column DNase I treatment (Qiagen) at 37 °C for 30 min in order to remove any contaminating genomic DNA. DNase I was removed with RNeasy mini kit according to the clean-up procedure. All samples were analyzed by using polymerase chain reaction (PCR) with primers SN80f and SN80r **(**Additional file 3: Table S3**)**, in order to confirm the absence of genomic DNA contamination. The purity of RNA samples was evaluated based on RIN value by using Agilent Bioanalyzer 2100 (Agilent technologies, Santa Clara, CA, US). The concentration of RNA samples was determined by NanoDrop ND-2000. The RNA samples with optimal quantity and purity were selected for next generation RNA-sequencing after cDNA synthesis.

### Library construction and data analysis

The strand-specific library was constructed using TruSeq® Stranded Total RNA Sample Preparation kit (Illumina, USA). The ribosomal RNA was removed and the RNA fragments were cleaved, while the first and second strand cDNA were synthesized. A single ‘A’ nucleotide was added to the 3′ ends of the blunt DNA fragments with repaired ends, and subsequently connected with adapters. The purified libraries were quantified using Qubit® 2.0 Fluorometer (Life Technologies, USA) and validated by Agilent 2100 bioanalyzer (Agilent Technologies, USA), in order to measure the concentration and confirm the insert size. The cluster was generated by cBot and RNA sequencing was performed using a 150 bp pair-end strategy with the Illumina Hiseq X10 platform (Illumina, USA) to generate 3 billion bases per sample. Thereafter, raw data was acquired, followed by pretreatment using Septk1. The read length was > 90 nt. The number of average clean reads, clean reads ratio and mapping ratio for WT and V_B1_-treated samples were 3.3G, 97.1 and 99.6%, and 3.2G, 97.14 and 99.4%, respectively. We deposited the raw data to GEO repository under accession number GSE114949.

The raw sequencing data were filtered to remove rRNA reads, sequencing adapters, short-fragment reads and other low-quality reads. The cleaned paired-end reads were mapped to *M.bovis* BCG reference genome with accession number AP010918 by using Tophat v2.0.9 with two mismatches [121]. After the completion of genome mapping, Cufflinks v2.1.1 [122] was run with a reference annotation, in order to generate FPKM (Fragments Per Kilobase of exon model per Million mapped reads) values for known gene models. Cuffdiff analysis was performed to identify the differentially expressed transcripts and genes [118]. False discovery rate (FDR) was used to determine the threshold of *P*-value in multiple tests, on the basis of FDR ≤0.05 and fold-change ≥2 [123, 124]. Furthermore, the homology of genes in *M. bovis* BCG was compared with *M. tuberculosis H37Rv* genome, by using BLAST analysis (https://blast.ncbi.nlm.nih.gov/Blast.cgi; Additional file 7: Table S2).

### cDNA synthesis and qRT-PCR

cDNA was synthesized using SuperScript III First-Strand Synthesis kit according to the manufacturer’s instructions. Quantitative real-time PCR (qRT-PCR) was performed with iQ™ SYBR Green Supermix (Bio-Rad) by using the LightCycler 480 II (Roche) RT-PCR System. The PCR conditions were as follows: initial denaturation at 95 °C for 3 min, followed by 40 cycles of denaturation at 95 °C for 10 s, annealing at 55 °C for 30 s and extension at 72 °C for 10 s. After completion of the 40th cycle, a final cycle of 50 °C was conducted for 3 min. The reactions were carried out with 50-fold diluted cDNA and 12.5 μL SYBR Green supermix. All reactions were carried out in triplicate, including the cDNA-free template and RT-free control. The *Rv2703* gene encoding RNA polymerase sigma A factor (*sigA*) was selected as a housekeeping gene for normalization of target gene expression levels. PCR reaction efficiency was calculated by evaluating the slope of a standard curve. The curve was created by linear regression of the resulting C_q_ values, with a 10-fold serial dilution of DNA. The 2^△△Cq^ method was used to calculate the relative expression levels of target genes.

### LC-MS based metabolomic analysis

The BCG strains cultured with 7H9 medium in the absence and/or presence of V_B1_ were collected after 48 h incubation at 37 °C. In the collected 60 mg pellets, added 500 μL of methanol (pre-cooled at − 20 °C) and 500 μL deionized water (ddH_2_O) (4 °C) and vortex for 30 s, then added 100 mg glass beads. Placed the tubes into liquid nitrogen for 5 min and thawed at roomtemperature, then put tubes in the high flux organization grinding apparatus, 70 Hz for 2 min, repeated twice, centrifuged for 10 min at 13000 g (4 °C), and transfered supernatant into a new centrifuge tube. Samples were blow-dried by vacuum concentration. Chromatographic separation was carried out in the Shimadzu LC-30A system equipped with ACQUITY UPLC® HSS T3 (150 × 2.1 mm, 1.8 μm, Waters) column, with a column temperature of 40 °C. Gradient elution of the analytes was carried out with solvent A containing 0.1% formic acid in water and solvent B containing acetonitrile, at a flow rate of 0.3 mL/min. After equilibration, 5 μL of sample was injected into LC column, followed by an increasing linear gradient of solvent B (v/v): 0~0.5 min, 2% B; 0.5~9 min, 2%~ 50% B; 9~12 min, 50%~ 98% B; 12~13 min, 98% B; 13~14 min, 98%~ 2% B; 14~15 min, 2%.

Mass spectrometry (MS) analysis was executed in an electrospray ionization mode by using AB SCIEX TripleTOF™ 5600 System. The optimization of the operating parameters were achieved as follows: spray voltage, 5500 V (positive), 4500 V (negative); nebulizer gas (NEB), 50 psi; auxiliary gas (AUX), 50 psi; curtain gas (CUR), 35 psi; source temperature, 500 °C. Full-scan MS analysis was performed over the mass range of m/z 100–1500, with a collision energy of 45 eV.

Metabolites were characterized by comparisons with reference standards or MS/MS fragment information obtained from the Human Metabolome Database (HMDB) (http://www.hmdb.ca), the Metlin Database (http://metlin.scripps.edu), the massbank Database (http://www.massbank.jp/), the LipidMaps Database (http://www.lipidmaps.org) or the mzclound Database (https://www.mzcloud.org). We used IDA (Information Dependant Acquistion) and selected the signal intensity of the first 8 ions in the parent ion for secondary fragmentation (Additional file 8: Figure S1).

### UPLC-MS (high resolution ultra-performance liquid chromatography-mass spectrometry) determination of amino acids

An equimolar standard mixture of all 20 amino acids(L-glycine, L-sarcosine, L-alanine, L-valine, L-proline, L-threonine, L-isoleucine, L-leucine, L-ornithine, L-methionine, L-histidine, L-phenylalanine, L-arginine, L-tyrosine, L-aspartic acid, L-tryptophan, 4-aminobutyric acid, L-serine, L-lysine, L-glutamic acid) was prepared, the final concentration was 0.2 μg/mL, 0.5 μg/mL, 1 μg/mL, 2 μg/mL, 5 μg/mL, 10 μg/mL, 20 μg/mL and 50 μg/mL.

100 mg of bacteria pellets was ground into fine powder in liquid nitrogen, amino acids were extracted with 1 mL HCl overnight. The tubes were centrifuged at 12000 rpm for 5 min to collecte the supernatant. 100 μL of isotope-labelled amino acids (Alanine -d4) internal standard was then added to the tube and blended, and drying with a moderate nitrogen gas stream. For derivatisation, 60 μL of derivatisation reagent [hydrochloric acid/n-butyl alcohol (1: 3, v/v)] was added into the mixture and reacted for 15 min at 65 °C. Then, the sample was separated and analysed by UPLC-MS.

The ACQUITY UPLC System equipped with a 1.7 μm C18 column (ACQUITY UPLC BEH, 2.1 × 100 mm, Waters) set at 40 °C was used for chromatographic separation. Gradient elution of the analytes was implemented with solvent A containing 0.1% formic acid and 0.1% heptafluorobutyric acid in acetonitrile, solvent B containing 0.1% formic acid in water, at a flow rate of 0.25 mL/min. Five microliter of sample was injected following equilibration. An increasing linear gradient of solvent A (v/v) was used as follows: 0~1.5 min, 5% A; 1.5~2 min, 5~20% A; 2~7 min, 20~30% A; 7~8.5 min, 30~98% A; 8.5~10.5 min, 98% A; 10.5~11 min, 98~5% A; 11~12.5 min, 5% A.

The ESI source was used in positive mode by multiple reaction monitoring (MRM) mode. The following conditions of detection were applied: ion source capillary voltage, 3200 V; cone voltage, 20 V; desolvation temperature, 380 °C.

### Statistical analysis

All experiments were performed at least in biological triplicate and the results are presented as the mean ± standard deviation (SD). Values were considered to be statistically significant when *P*-value was < 0.05 and the statistical significance of the observed differences was assessed with one way analysis of variance (one-way ANOVA).

## Additional files

Additional file 1: **Table S1.** The complete list of differentially expressed genes in control and V_B1_-treated samples. (XLS 111 kb)

Additional file 2: **Table S2.** The change fold of genes listed in the manuscript. (XLSX 11 kb)

Additional file 3: **Table S3.** The primers used in this study. (DOCX 22 kb)

Additional file 4: **Table S4.** The complete list of differentially produced metabolites in control and V_B1_-treated samples. (XLSX 26 kb)

Additional file 5: **Table S5.** The complete list of detected amino acids in control and V_B1_-treated samples. (XLSX 23 kb)

Additional file 6: **Table S6.** The associated KEGG analysis of transcriptomics and metabolomics in control and V_B1_-treated samples. (XLS 44 kb)

Additional file 7: **Table S7.** The homologous genes between *M. bovis* BCG and *M. tuberculosis H37Rv. (XLS 1840 kb)*

Additional file 8: **Figure S1.** Fragmentation spectrum for LC-MS. (DOCX 1592 kb)

## Abbreviations

ADP: Adenosine diphosphate
FDR: False discovery rate
GTP: Guanosine triphosphate
INH: Isoniazid
MDR: Multidrug resistant
MS: Mass spectrometry
Mtb: *Mycobacterium tuberculosis*
NAD: Adenine dinucleotide
OD: Optical density
PCR: Polymerase chain reaction
PFK: Phosphofructokinase
qRT-PCR: Quantitative real-time PCR
RIF: Rifampicin
SD: Standard deviation
TG: Triacylglycerol
TGS: Triacylglycerol synthase
ThDP: Thiamin diphosphate
ups: Universal stress proteins
V_A_: Vitamin A
V_B1_: Vitamin B_1_
Vc: Vitamin C
V_D_: Vitamin D
