# Supplementary material for: The prominent alteration in transcriptome and metabolome of Mycobacterium bovis BCG str. Tokyo 172 induced by vitamin B1
Source: BMC Microbiol. 2019 May 22;19:104. doi: 10.1186/s12866-019-1492-9 (PMC6530141; doi:10.1186/s12866-019-1492-9)
Supplement: Supplementary file 3 — Table S3. The primers used in this study. (DOCX 22 kb) [file 12866_2019_1492_MOESM3_ESM.docx]

| **Primer name** | **Sequence (5’-3’)** | **Use** |
| --- | --- | --- |
| SN1 (f) | 5’-GGGAATTCCATATGAACGAGGCGCTCGACGAT-3’ | Primer for clone ORF of *Rv3291c* |
| SN1 (r) | 5’-CCCAAGCTTTGGTATATGCTGCCTATCG-3’ | Primer for clone ORF of *Rv3291c* |
| SN80 (f) | 5’-CGGAGAAGGTGCTGGAAATC-3’ | qRT-PCR for *sigA* |
| SN80 (r) | 5’-AAATCGCCAAGCTGGCTGTC-3’ | qRT-PCR for *sigA* |
| SN267 (f) | 5’-GGCAAGTGGCGGGTGGTGTTC-3’ | qRT-PCR for *JTY_RS12600* |
| SN267 (r) | 5’-GAGCATCGGGAAGGGTAACG-3’ | qRT-PCR for *JTY_RS12600* |
| SN268 (f) | 5’-TGATGGCAGAAGAGGTGATG-3’ | qRT-PCR for *JTY_RS07460* |
| SN268 (r) | 5’-GCGTCAGGTCGGTGGAAATG-3’ | qRT-PCR for *JTY_RS07460* |
| SN269 (f) | 5’-CAAGGCGGACTGGAAACACC-3’ | qRT-PCR for *JTY_RS15805* |
| SN269 (r) | 5’-TTCGTCGTCGTCCCAGTAGC-3’ | qRT-PCR for *JTY_RS15805* |
| SN270 (f) | 5’-AGTAACCGCCCTGTATGAG-3’ | qRT-PCR for *JTY_RS20155* |
| SN270 (r) | 5’-CGGCCTGATTCGGGAATTACGAC-3’ | qRT-PCR for *JTY_RS20155* |
| SN271 (f) | 5’-CCAAGGACGAGACCACCATC-3’ | qRT-PCR for *JTY_RS02300* |
| SN271 (r) | 5’-CGTAGTCGGAGTCGCTGTTC-3’ | qRT-PCR for *JTY_RS02300* |
| SN272 (f) | 5’-CCGGGTTCTGGCCCGATACTGG-3’ | qRT-PCR for *JTY_RS10910* |
| SN272 (r) | 5’-ATCAAAGCCTGCGACGACTG-3’ | qRT-PCR for *JTY_RS10910* |
| SN273 (f) | 5’-ACTTGACCGGGGCCAATTCG-3’ | qRT-PCR for *JTY_RS01000* |
| SN273 (r) | 5’-CGAACTACGAGGCTGGGACC-3’ | qRT-PCR for *JTY_RS01000* |
| SN274 (f) | 5’-ACGGTAAGAGTGGGAATCAG-3’ | qRT-PCR for *JTY_RS05235* |
| SN274 (r) | 5’-GAACCTCATCAGTGGCATAG-3’ | qRT-PCR for *JTY_RS05235* |
| SN275 (f) | 5’-TTCGGTTGCGGGTATCACGG-3’ | qRT-PCR for *JTY_RS18025* |
| SN275 (r) | 5’-TCGTTGTGGCGGATGGTGTC-3’ | qRT-PCR for *JTY_RS18025* |
| SN276 (f) | 5’-ACAACGGCTGGGACATCAAC-3’ | qRT-PCR for *JTY_RS00695* |
| SN276 (r) | 5’-CGGTGTAGAAACTGGATTGG-3’ | qRT-PCR for *JTY_RS00695* |
| SN277 (f) | 5’-TCGGTAGTGAGTGCGTGGATGTG-3’ | qRT-PCR for *JTY_RS10385* |
| SN277 (r) | 5’-CCAGATGCTGGTGTTGATCG-3’ | qRT-PCR for *JTY_RS10385* |
| SN278 (f) | 5’-GCTGGAAGACGAGATGAAAG-3’ | qRT-PCR for *JTY_RS10520* |
| SN278 (r) | 5’-ACCGCCACCGACACAGTAAG-3’ | qRT-PCR for *JTY_RS10520* |
| SN279 (f) | 5’-GCCGACCCGTTGCCACCGAC-3’ | qRT-PCR for *JTY_RS16215* |
| SN279 (r) | 5’-AGCACCGTTGTCGCAGTAGC-3’ | qRT-PCR for *JTY_RS16215* |
| SN280 (f) | 5’-GCTGTCCACGCTGCTGAAAC-3’ | qRT-PCR for *JTY_RS16230* |
| SN280 (r) | 5’-AGTGTGAGTCCGCGACGAACAG-3’ | qRT-PCR for *JTY_RS16230* |
| SN281 (f) | 5’-AGGTGGTGCGTCGTGGTCTG-3’ | qRT-PCR for *JTY_RS16225* |
| SN281 (r) | 5’-AGTTCAATGCCGTTGCCATC-3’ | qRT-PCR for *JTY_RS16225* |
| SN282 (f) | 5’-TTGACCTACACCGCCGAAG-3’ | qRT-PCR for *JTY_RS05530* |
| SN282 (r) | 5’-CGGCACTGTCATCGCATAGG-3’ | qRT-PCR for *JTY_RS05530* |
| SN283 (f) | 5’-CACAGCGGCACAACAATATGTC-3’ | qRT-PCR for *JTY_RS20045* |
| SN283 (r) | 5’-AACTGGTTTCGCACCGTGTC-3’ | qRT-PCR for *JTY_RS20045* |

Table S1-Primers used in this study

| **Primer name** | **Sequence (5’-3’)** | **Use** |
| --- | --- | --- |
| SN284 (f) | 5’-AGCGGGTGTCGTTGTTGTGC-3’ | qRT-PCR for *JTY_RS16220* |
| SN284 (r) | 5’-ATGGAGGTCACGGGCGATAC-3’ | qRT-PCR for *JTY_RS16220* |
| SN285 (f) | 5’-CCCTTACCGGCGGCGATAGC-3’ | qRT-PCR for *JTY_RS00455* |
| SN285 (r) | 5’-GCCCGAAATCTCCACCTCAC-3’ | qRT-PCR for *JTY_RS00455* |
| SN286 (f) | 5’-CGGCGGCGGTATCAATGTCG -3’ | qRT-PCR for *JTY_RS10510* |
| SN286 (r) | 5’-ACTCCCGCATCACCGAGCAG-3’ | qRT-PCR for *JTY_RS10510* |
