# Supplementary figures and images for: The prominent alteration in transcriptome and metabolome of Mycobacterium bovis BCG str. Tokyo 172 induced by vitamin B1
Source: BMC Microbiol. 2019 May 22;19:104. doi: 10.1186/s12866-019-1492-9 (PMC6530141; doi:10.1186/s12866-019-1492-9)

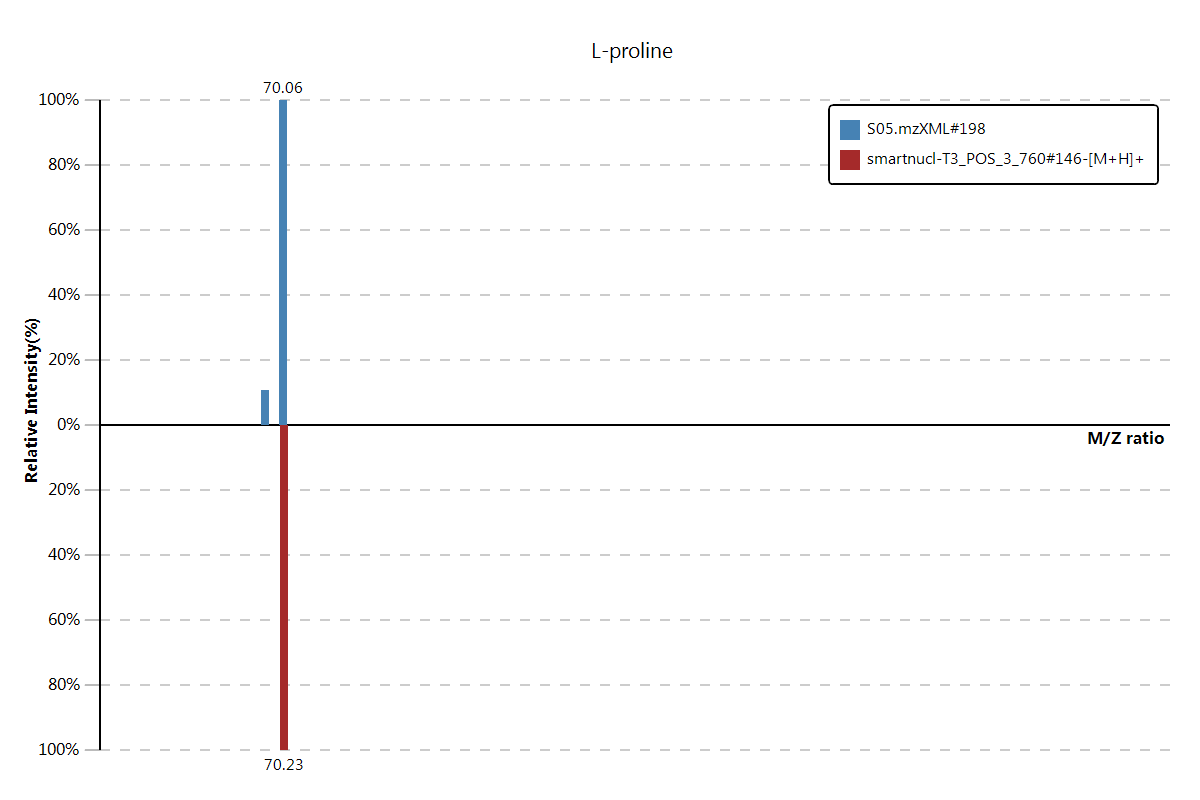


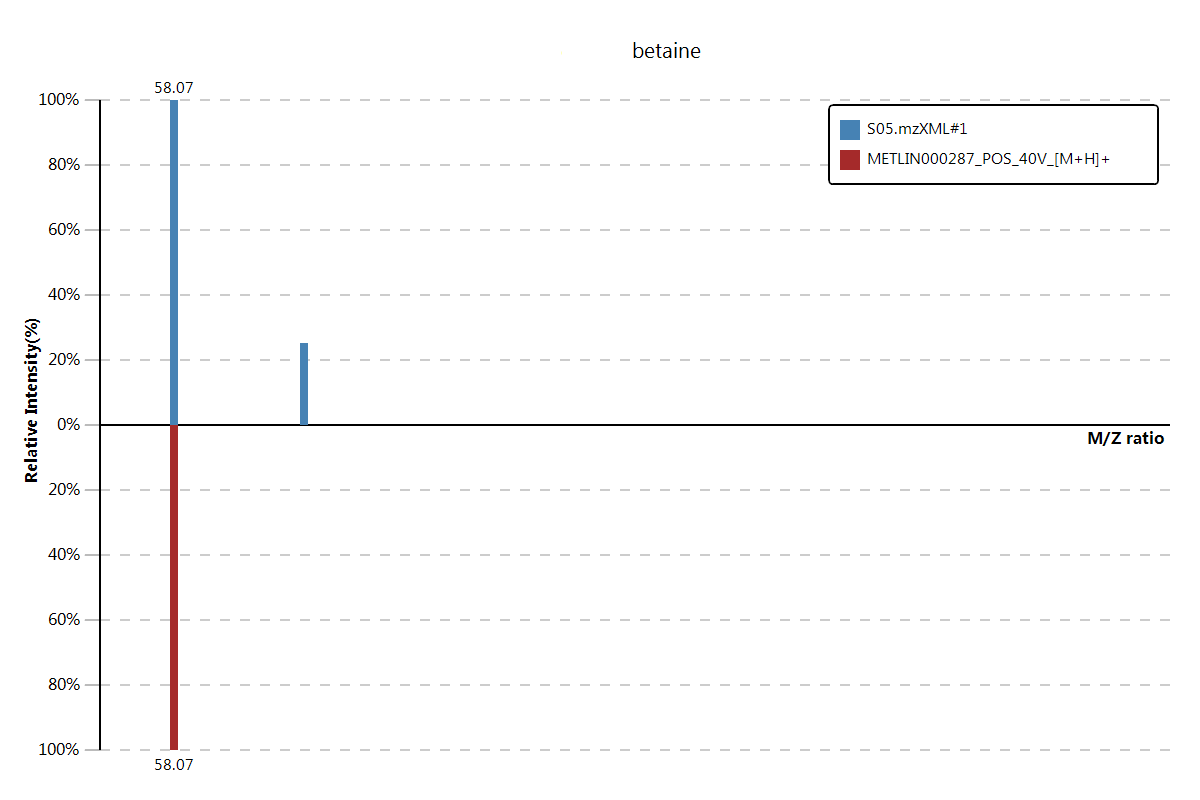


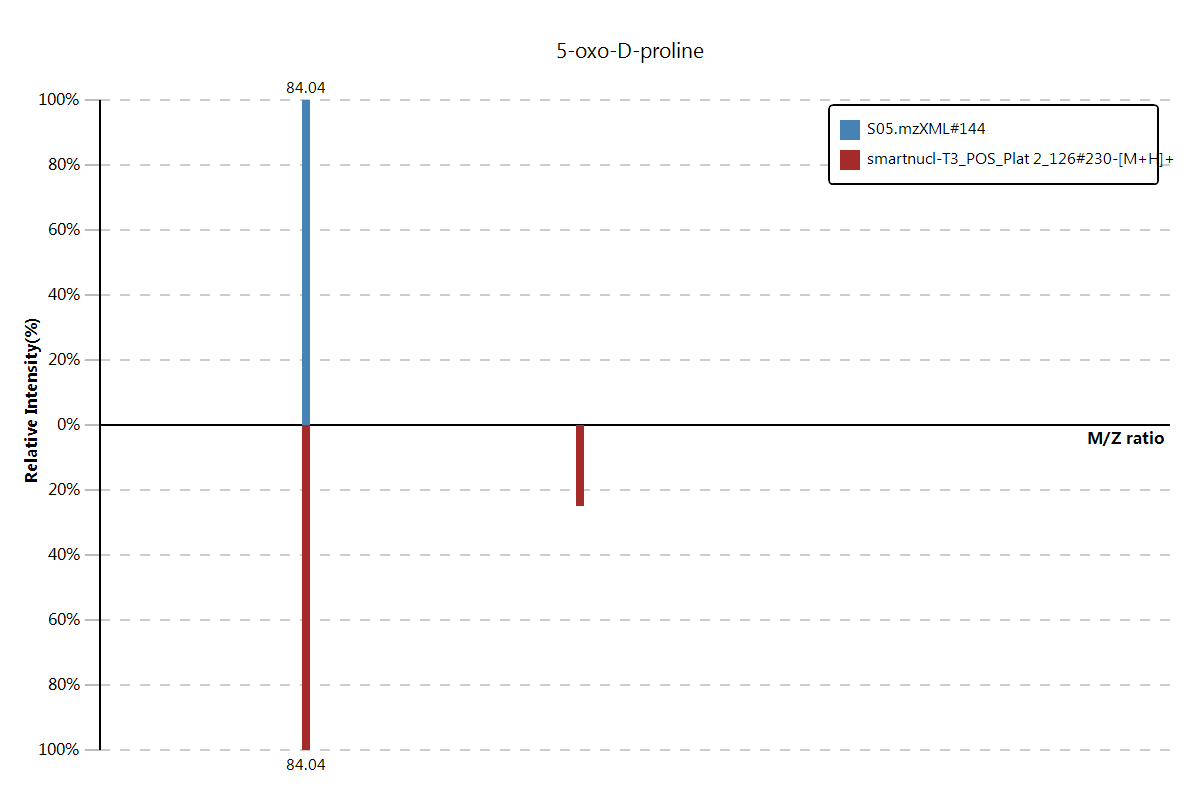


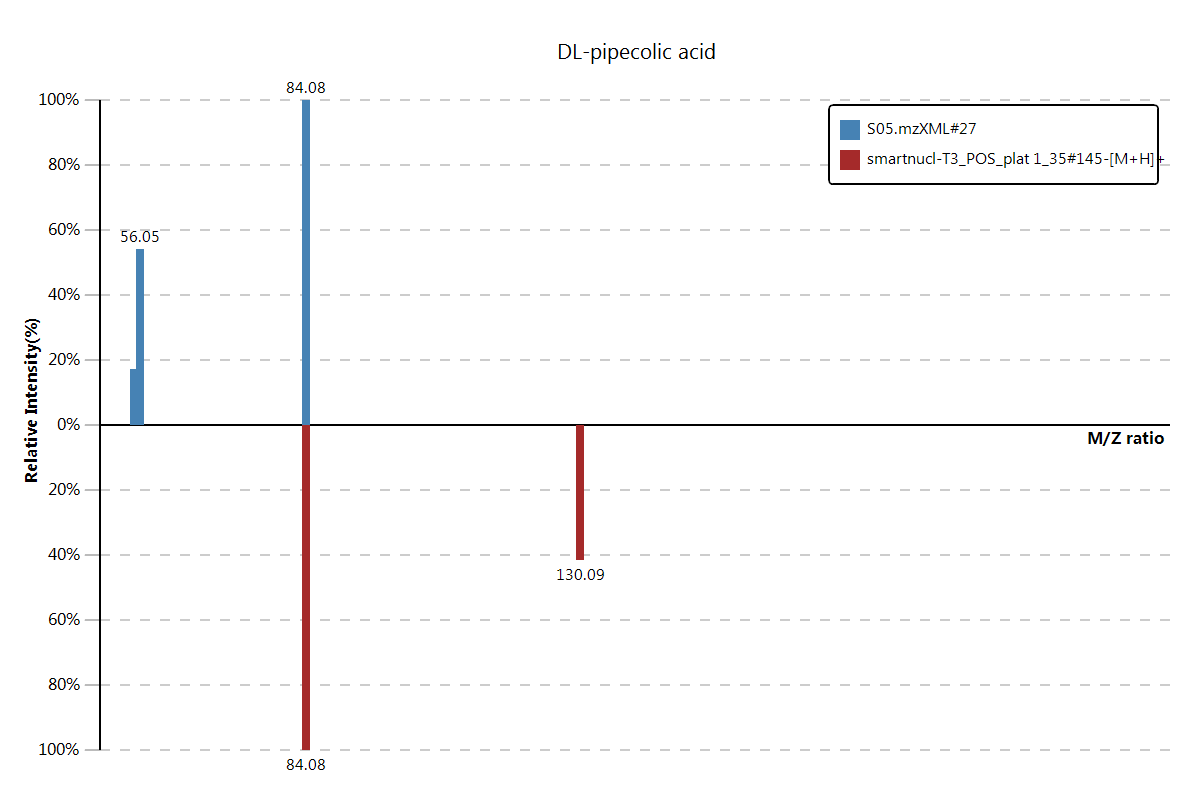


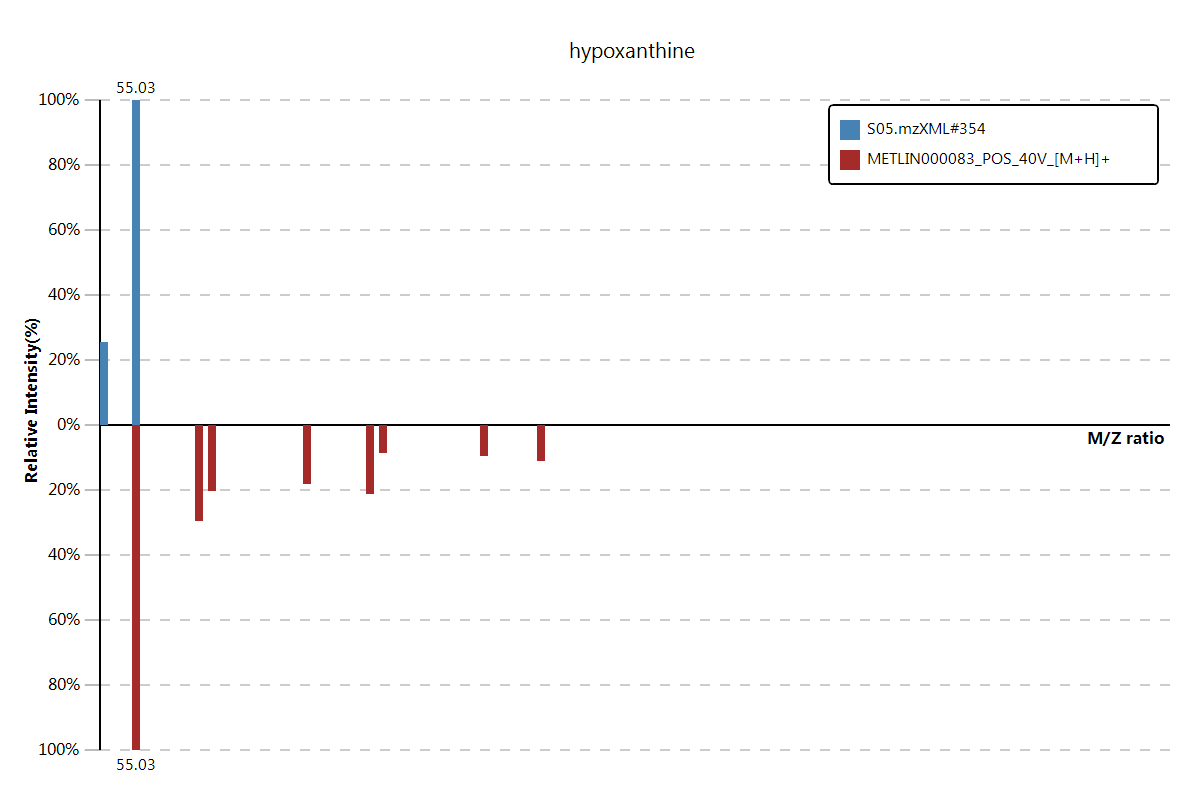


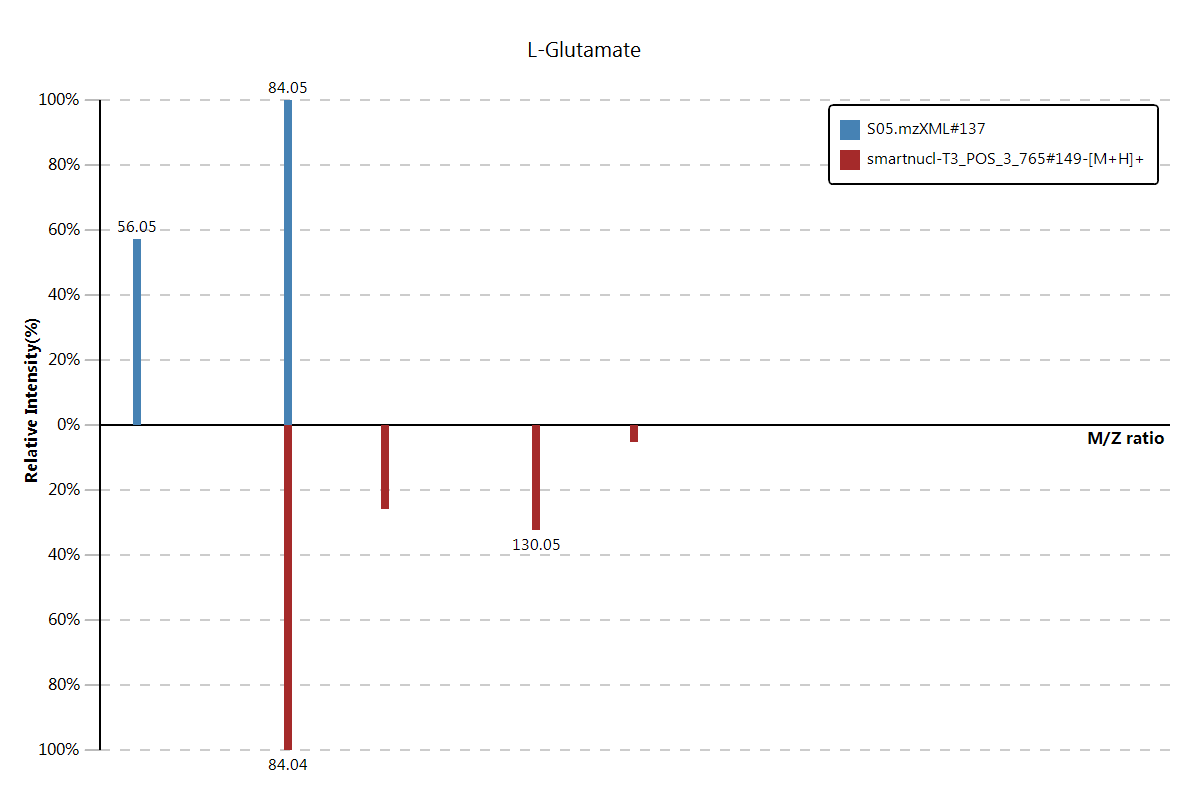


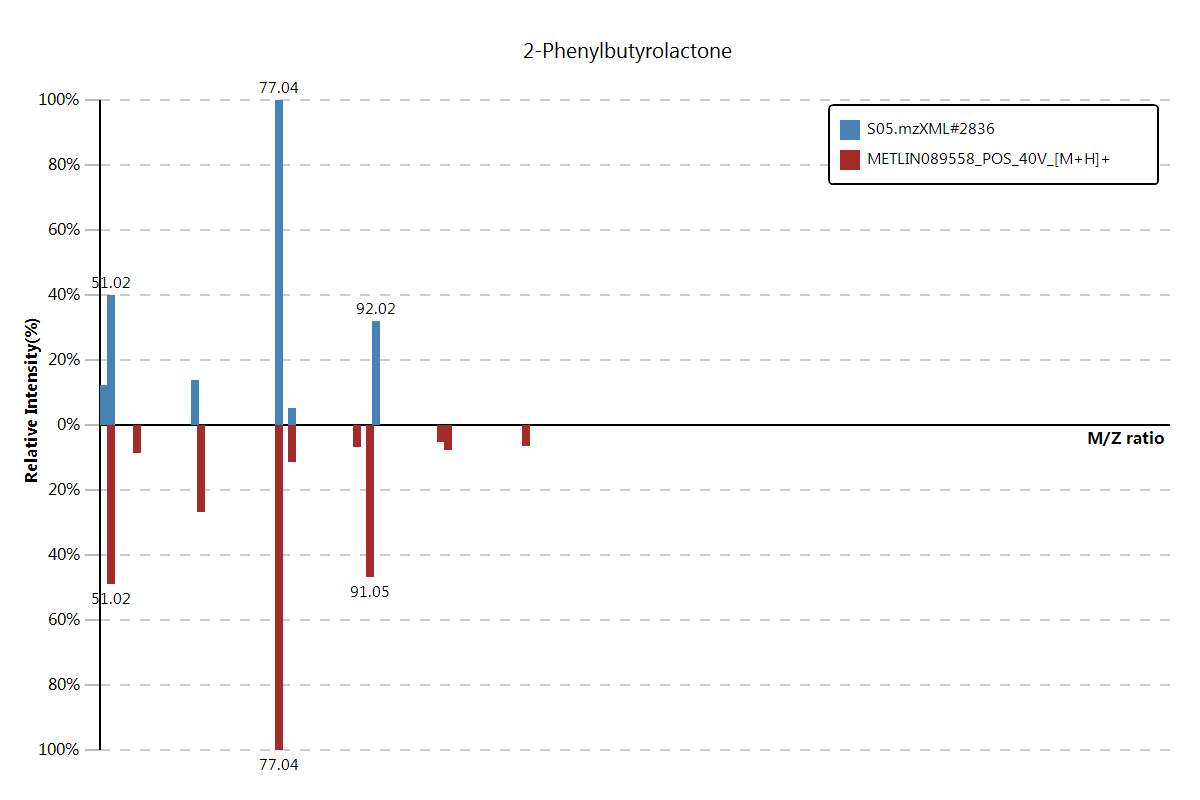


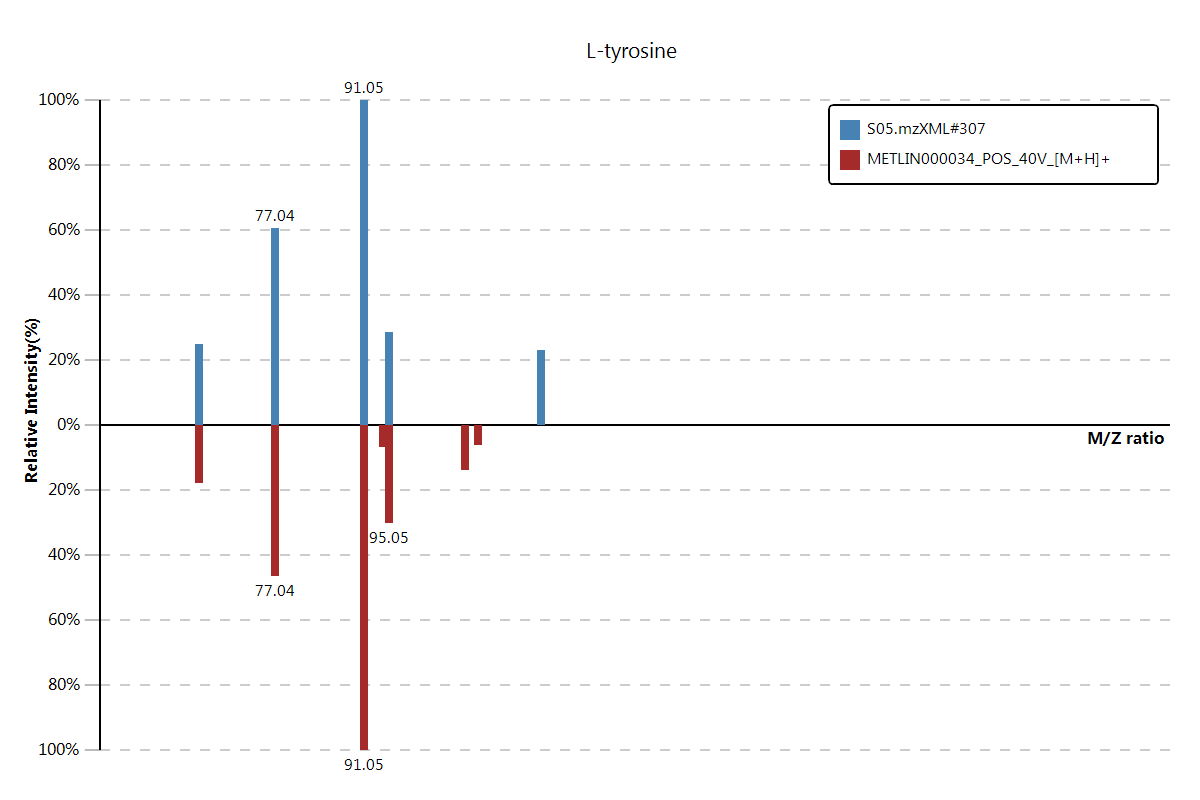


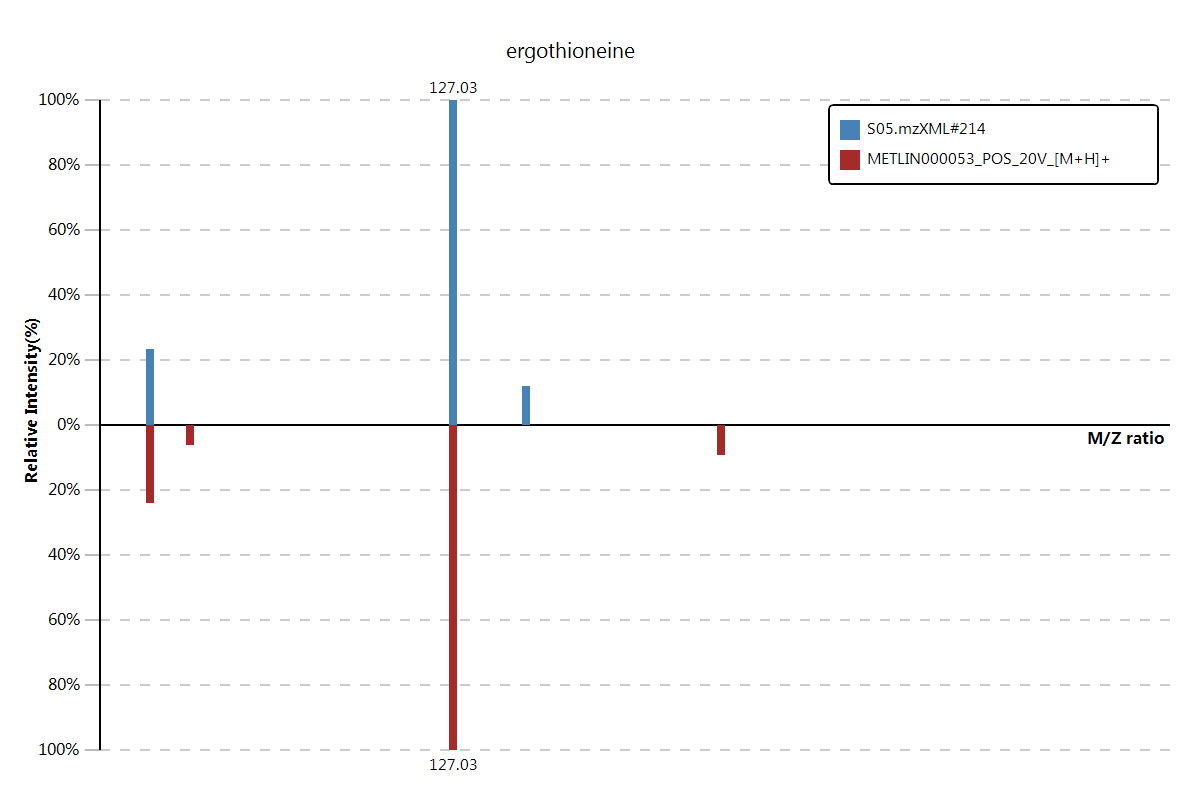


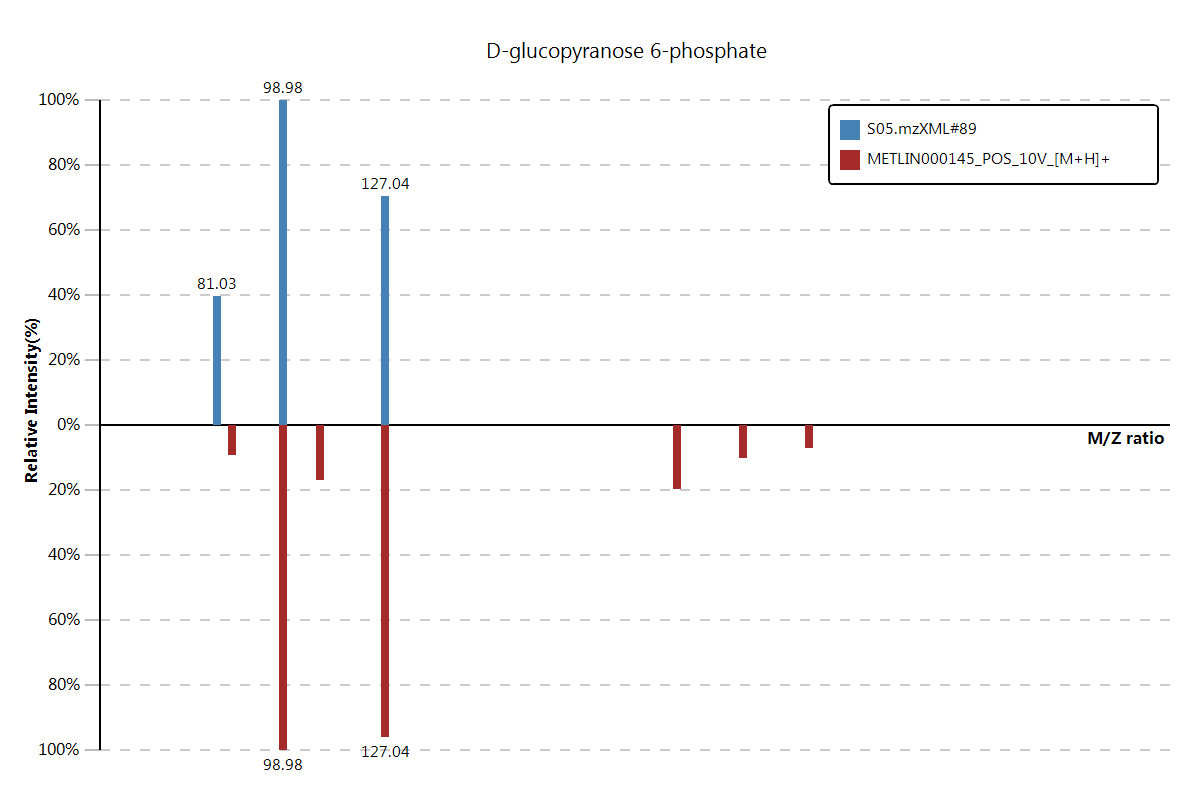


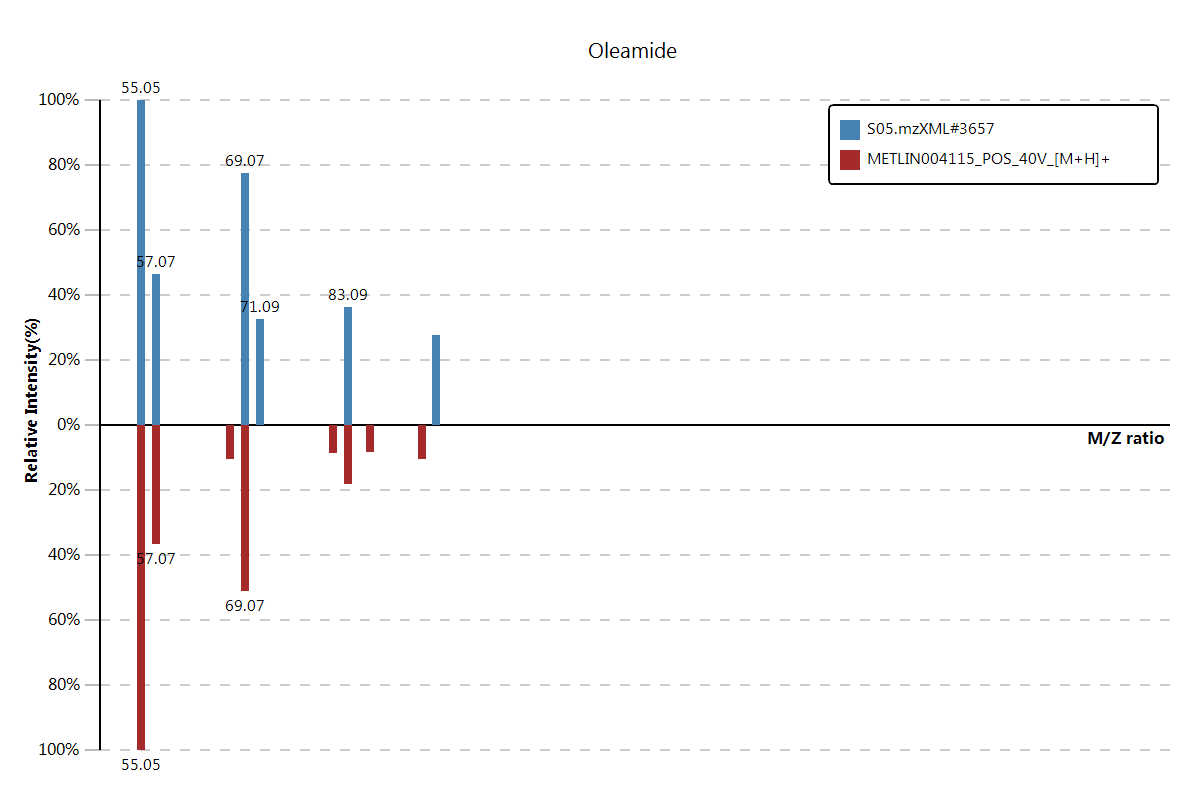


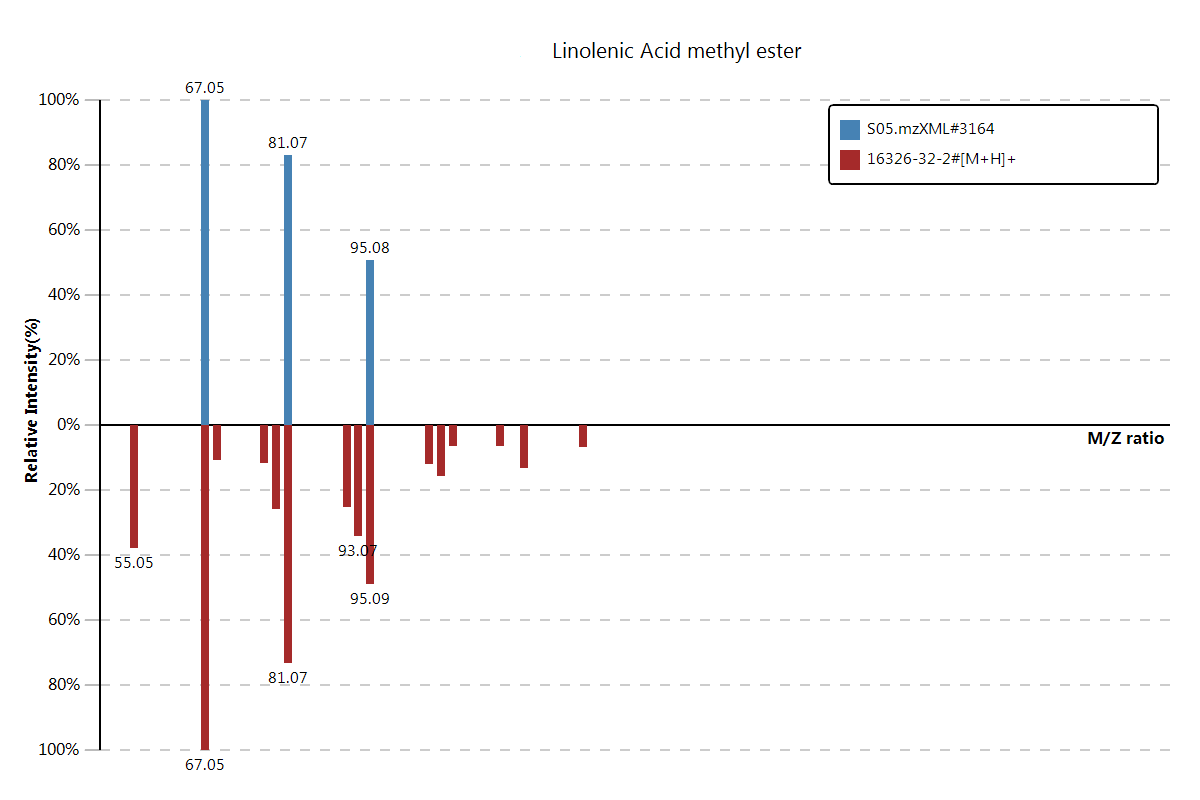


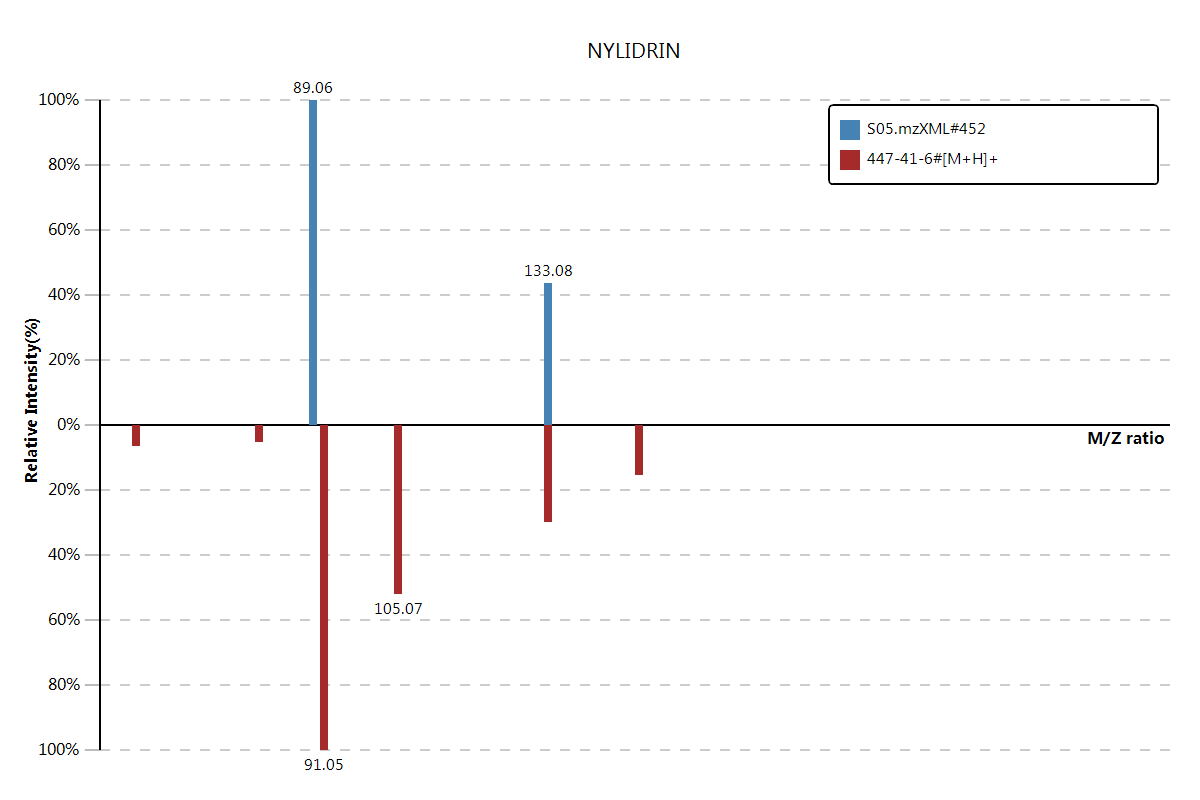


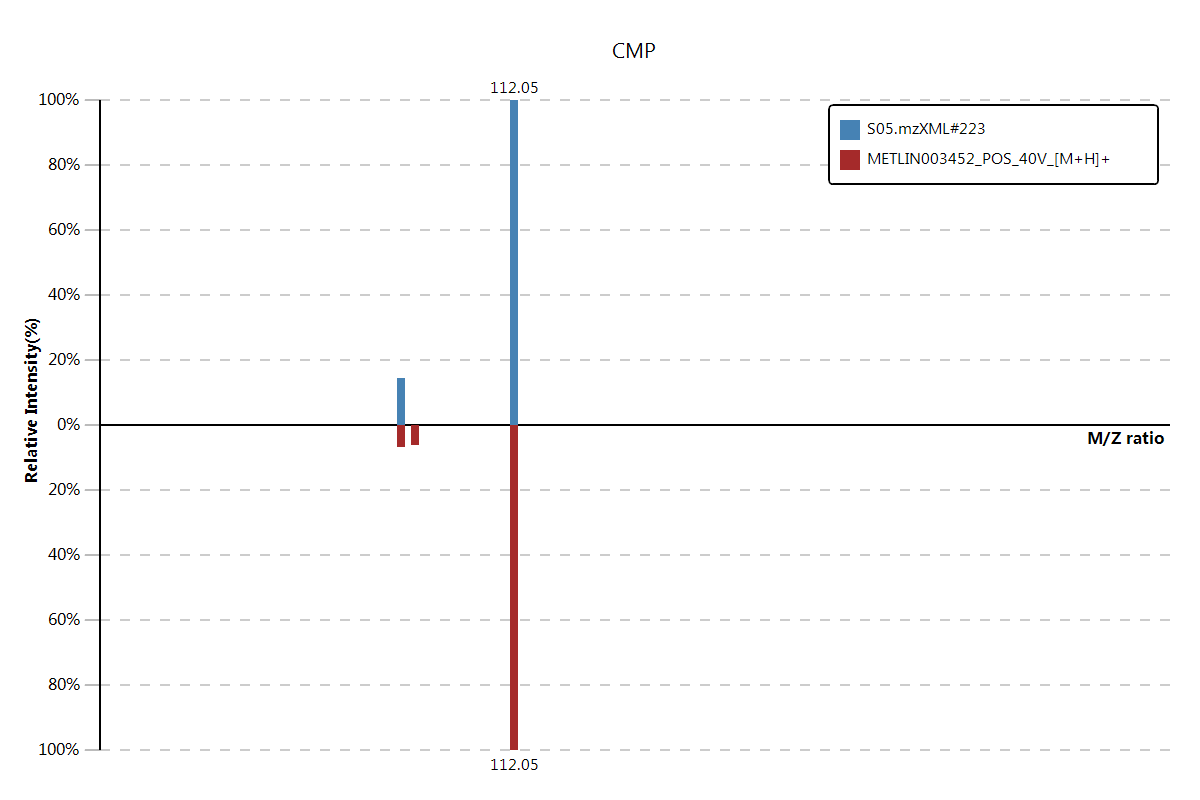


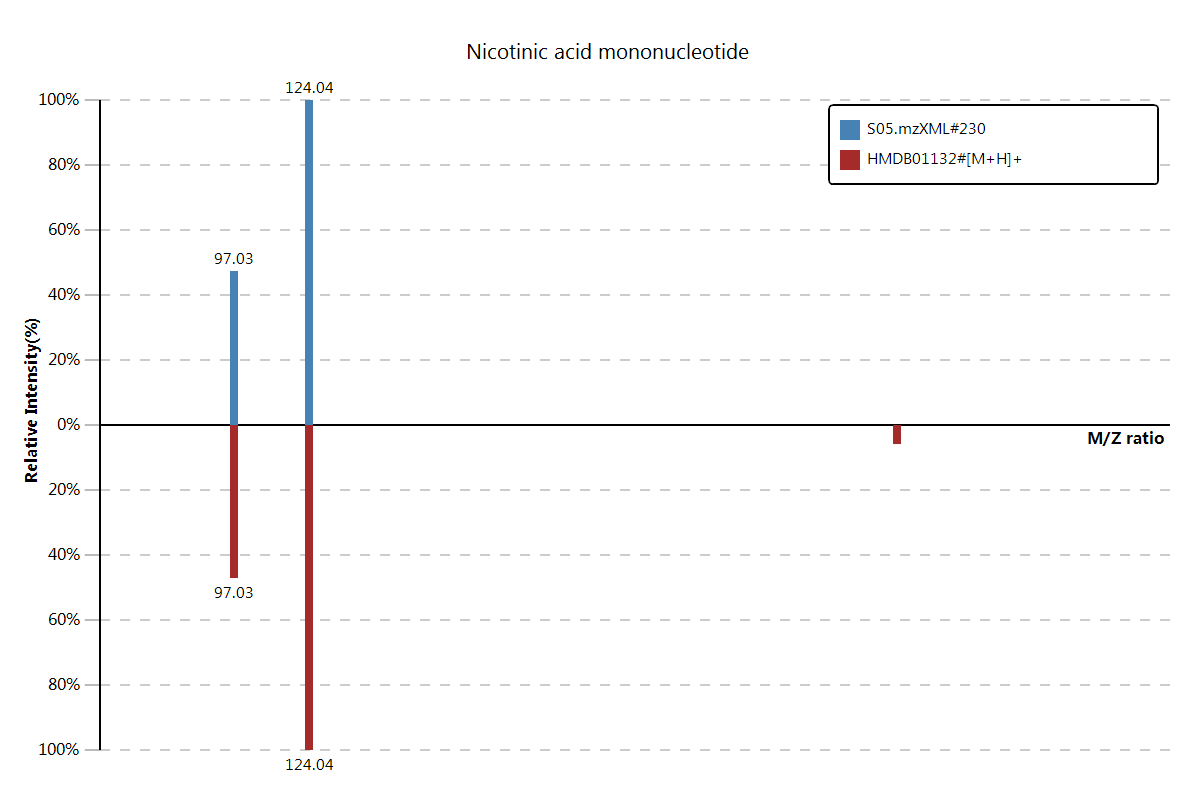


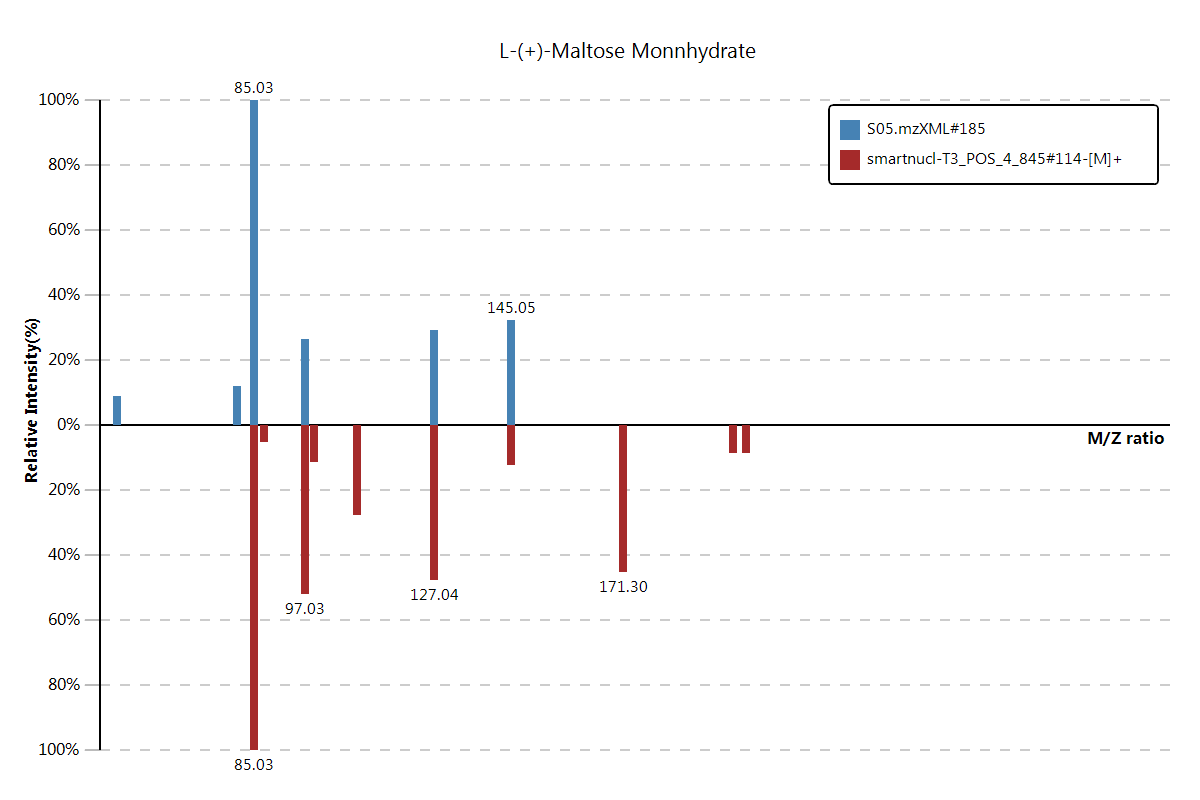


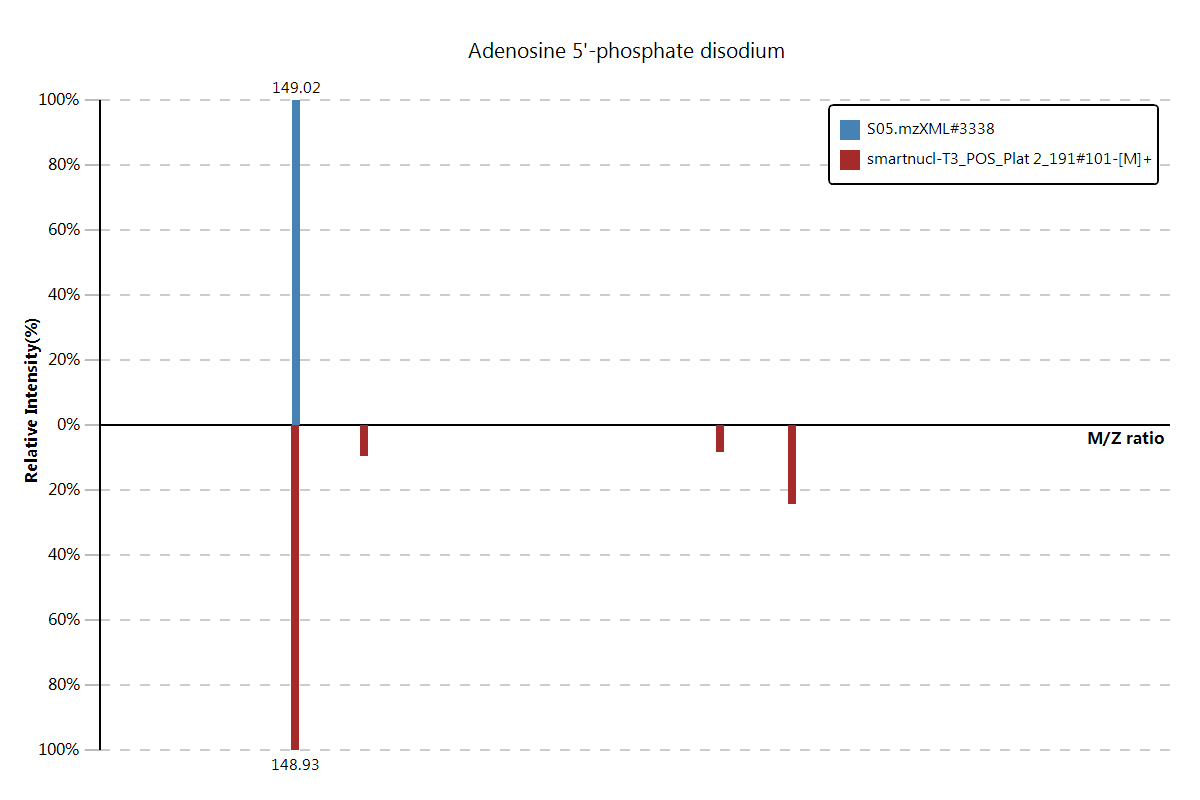


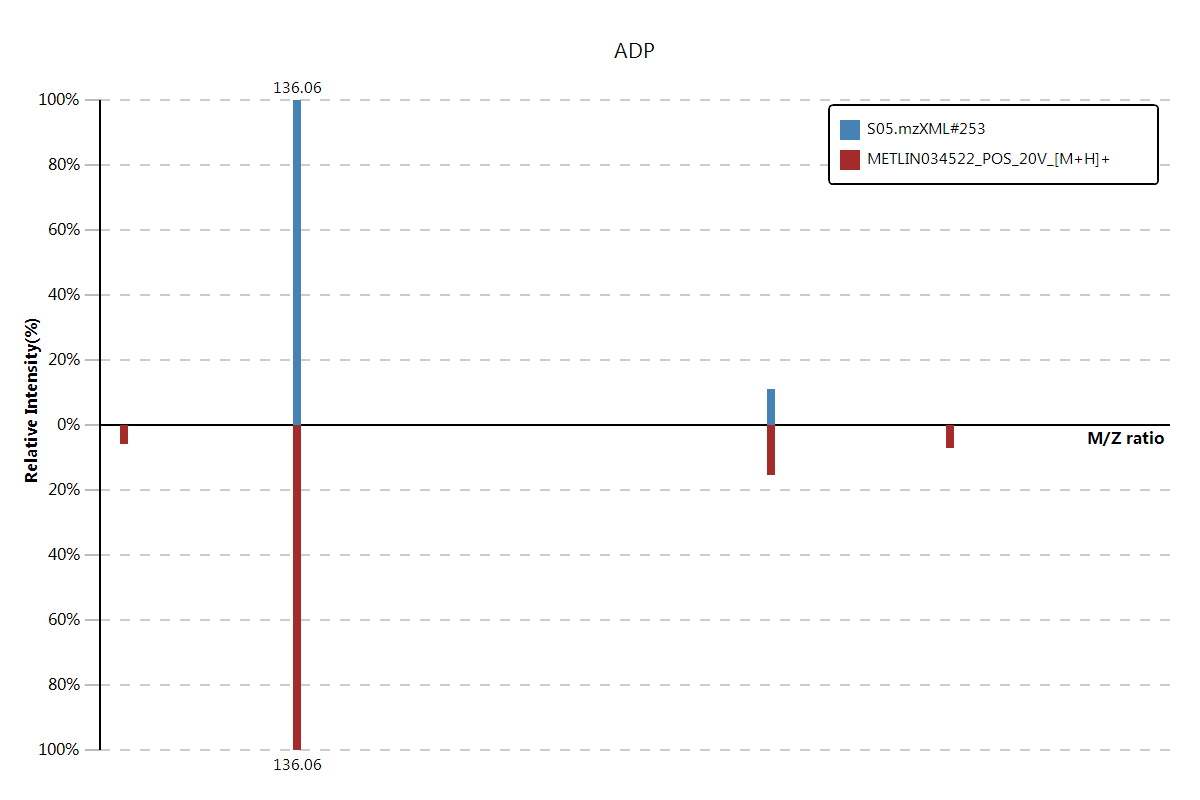


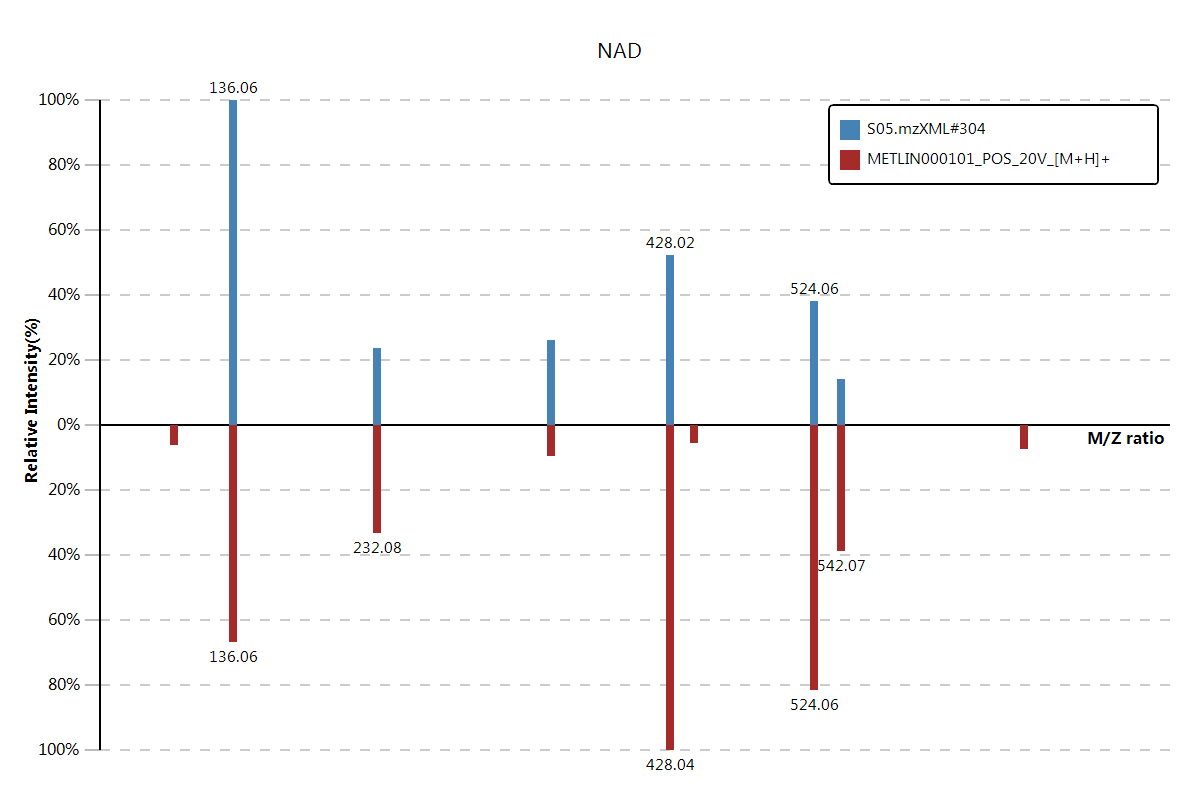


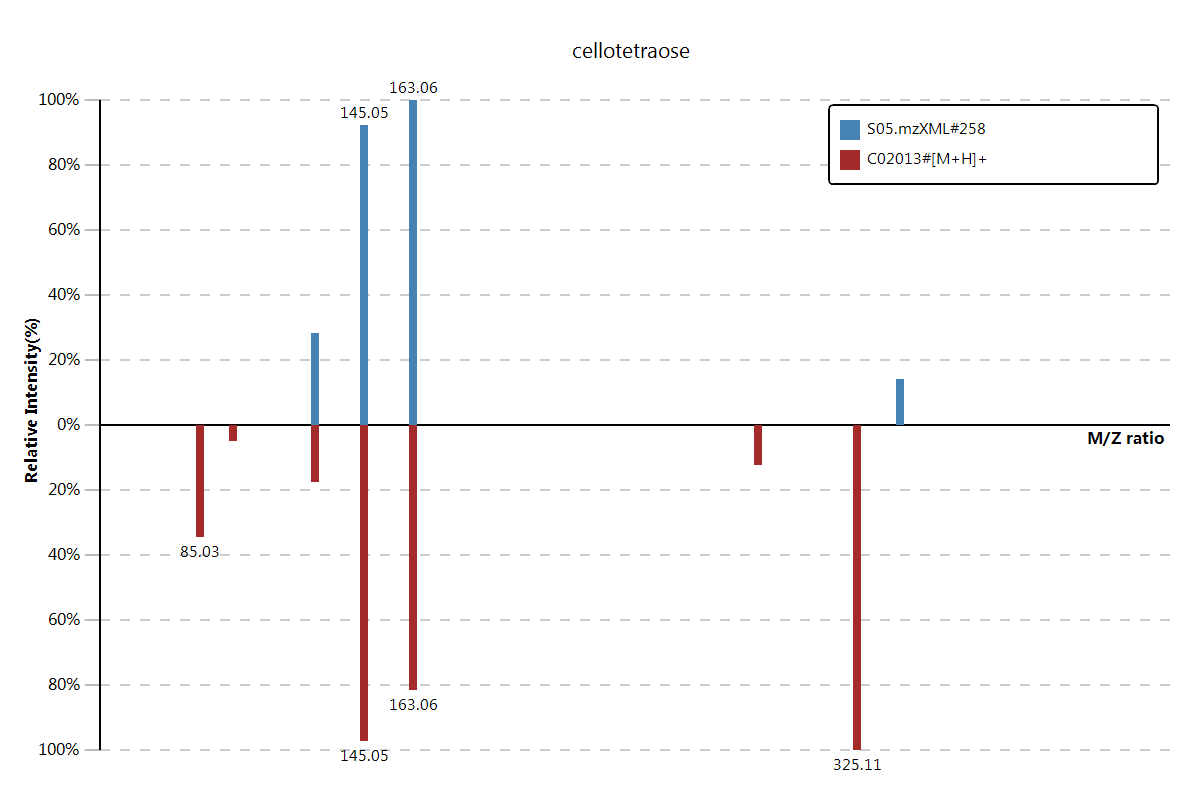


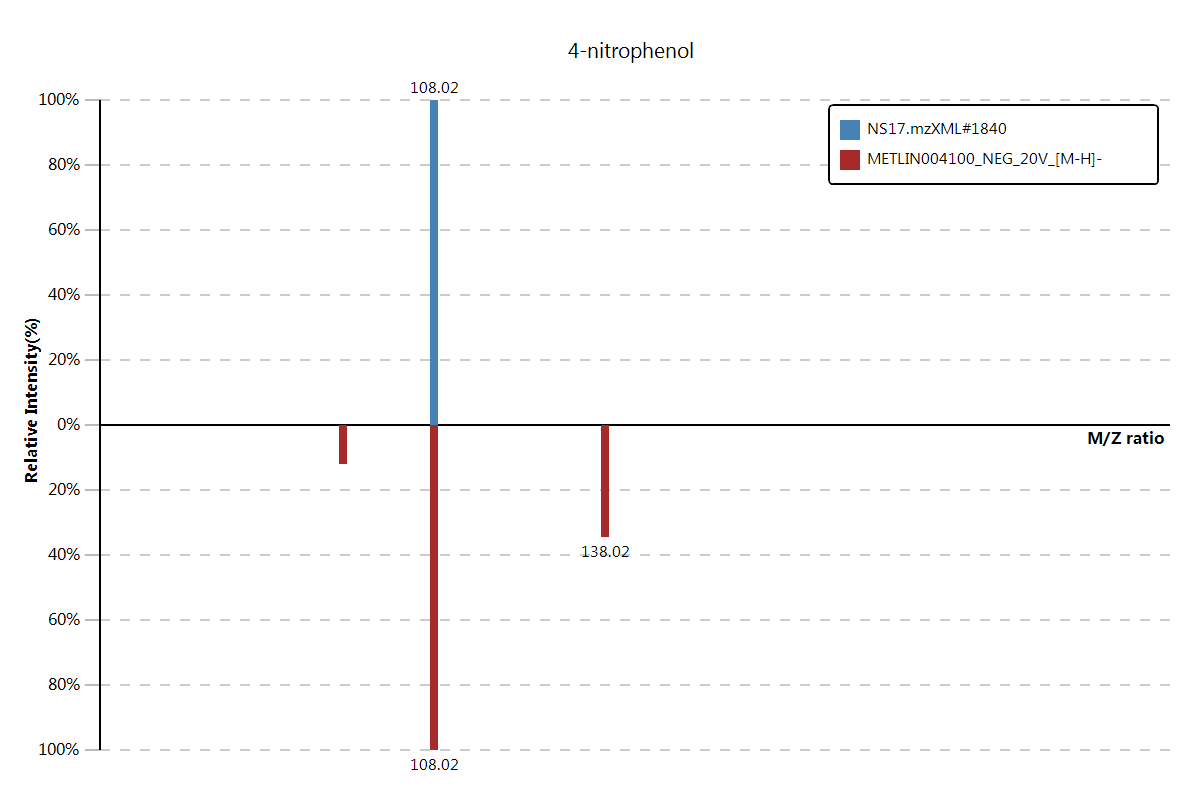


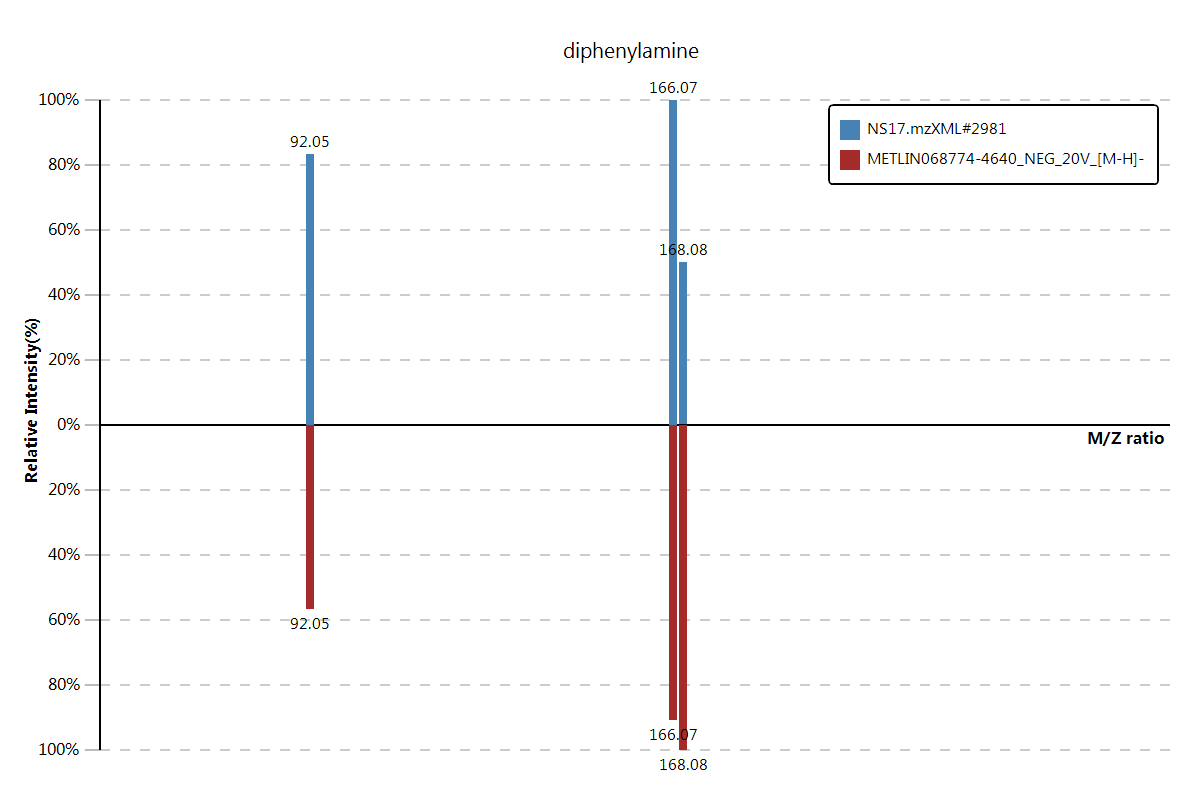


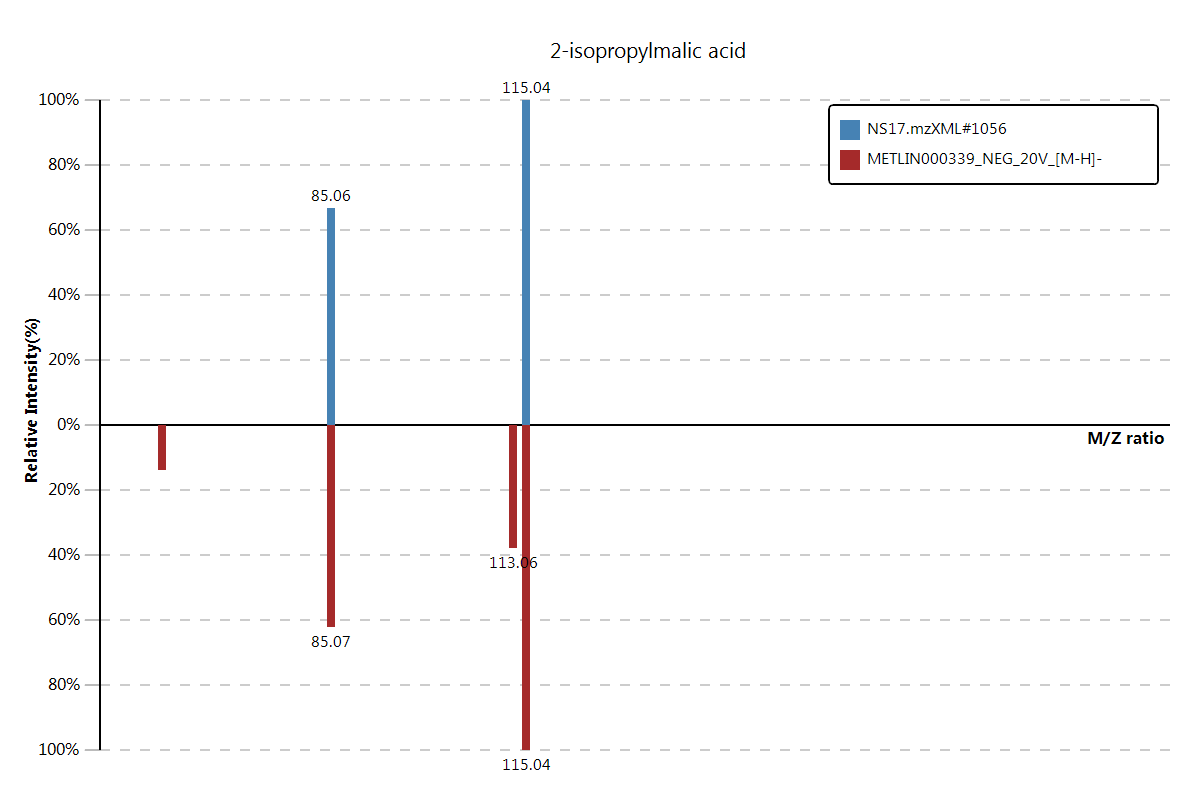


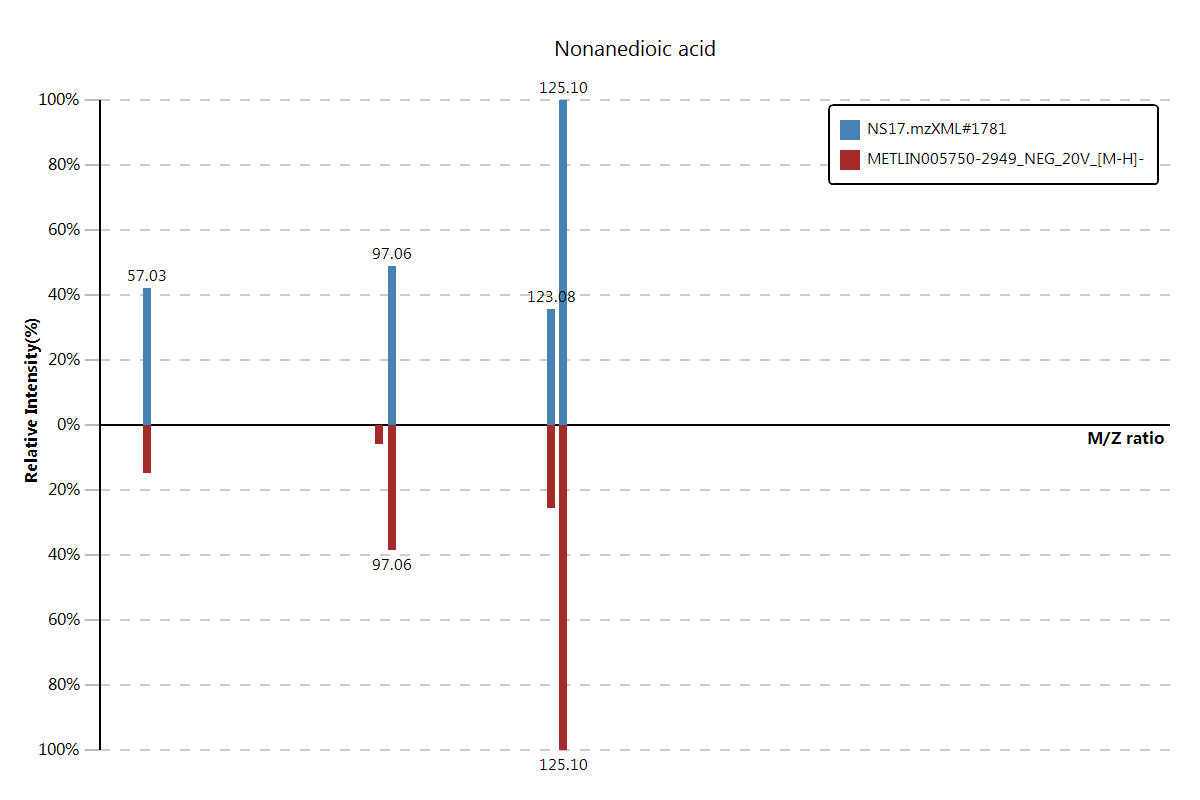


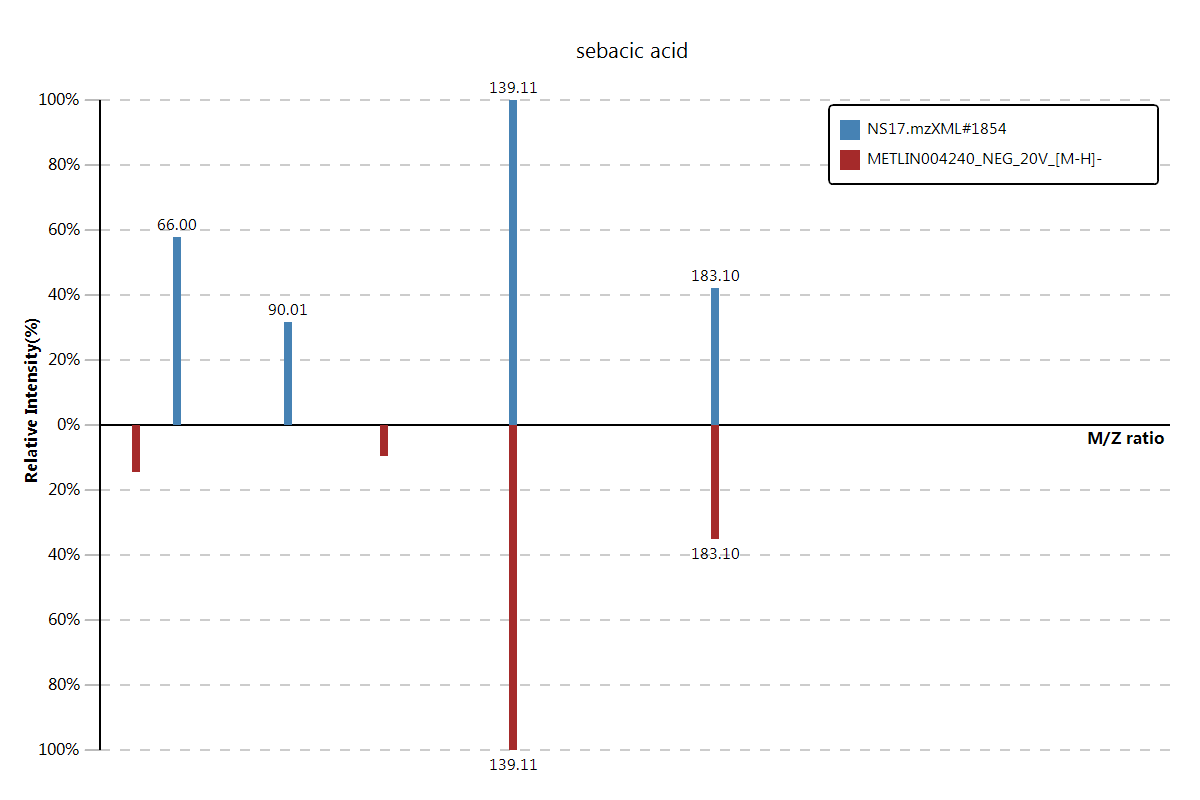


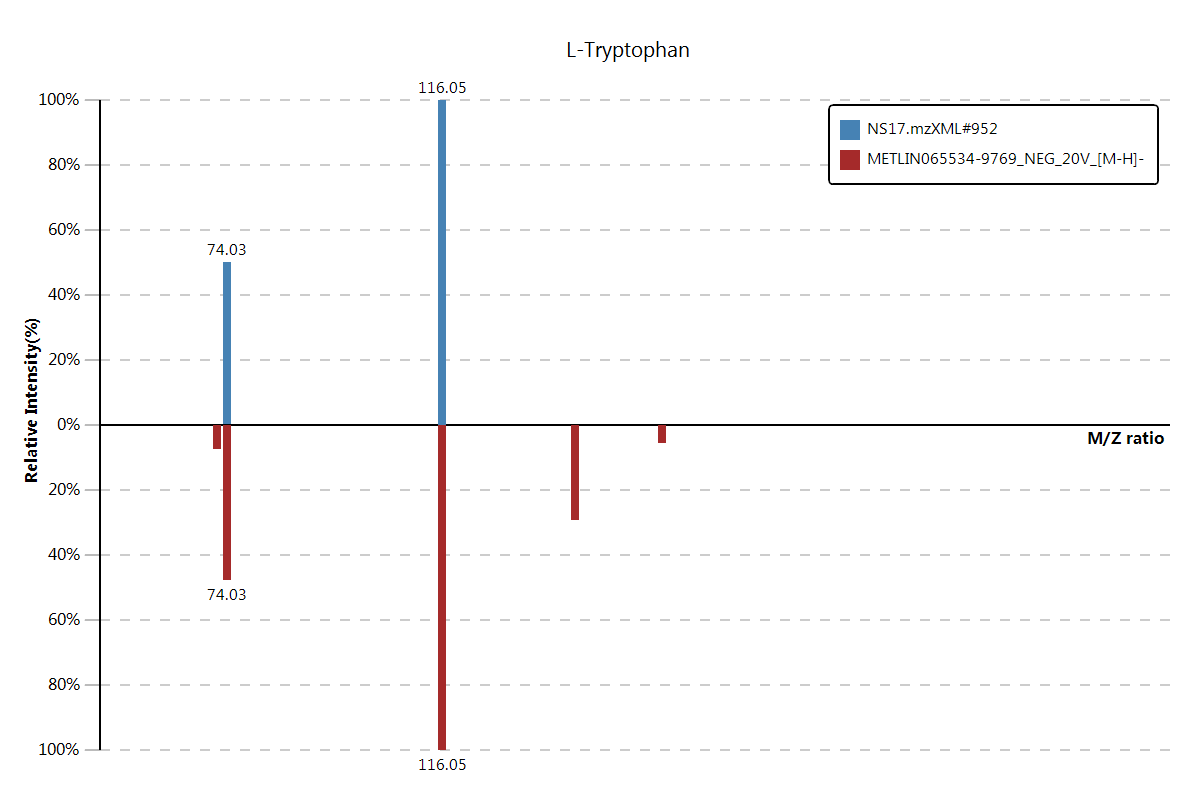


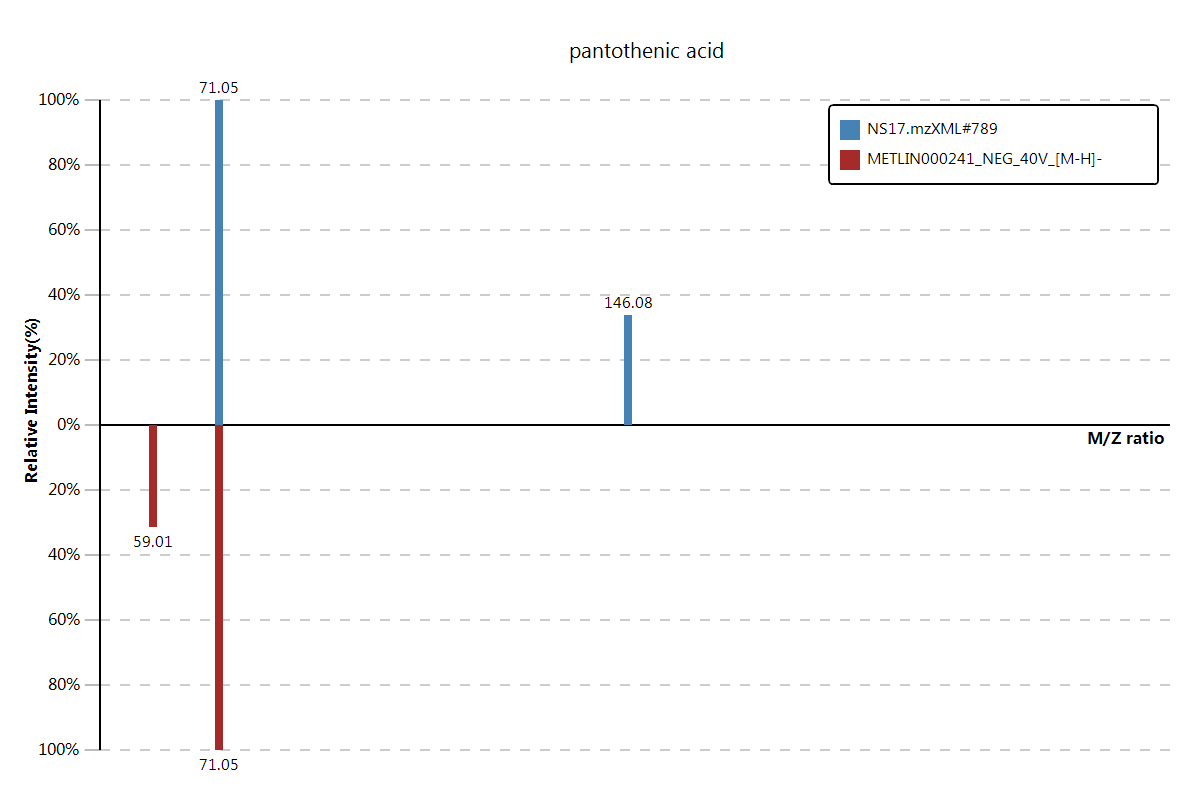


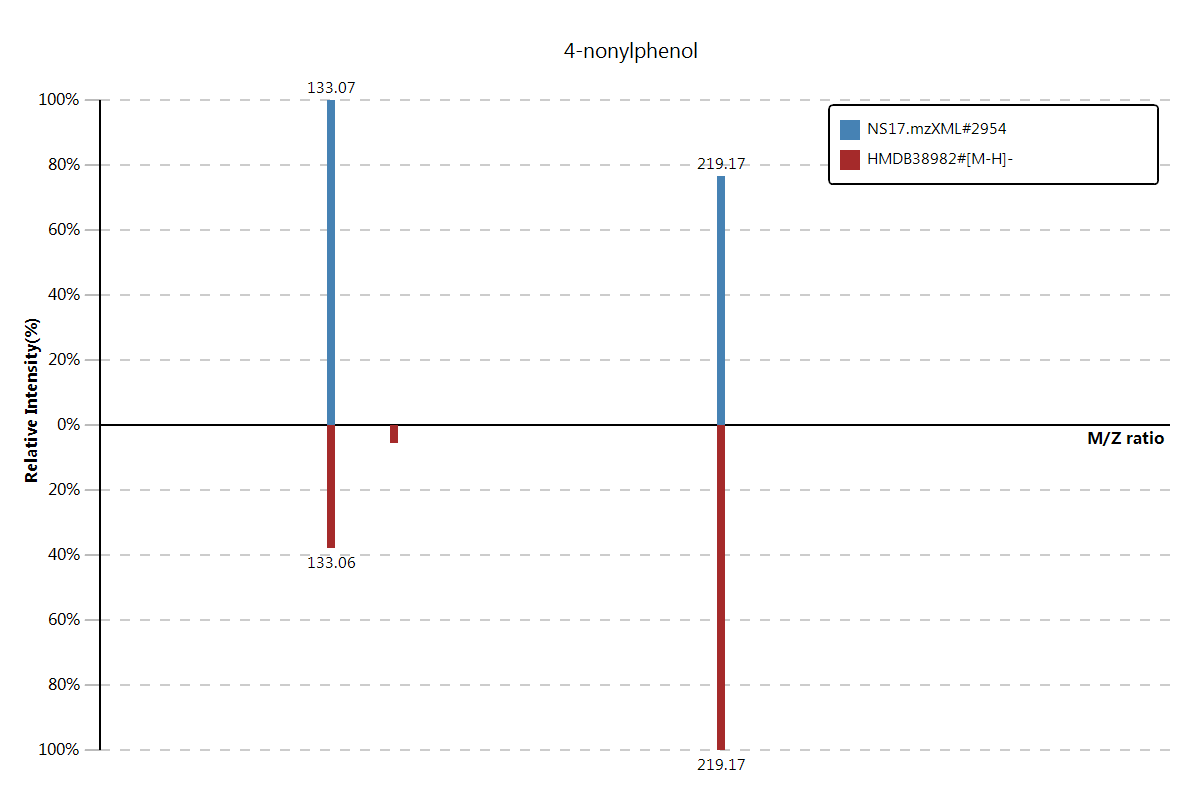


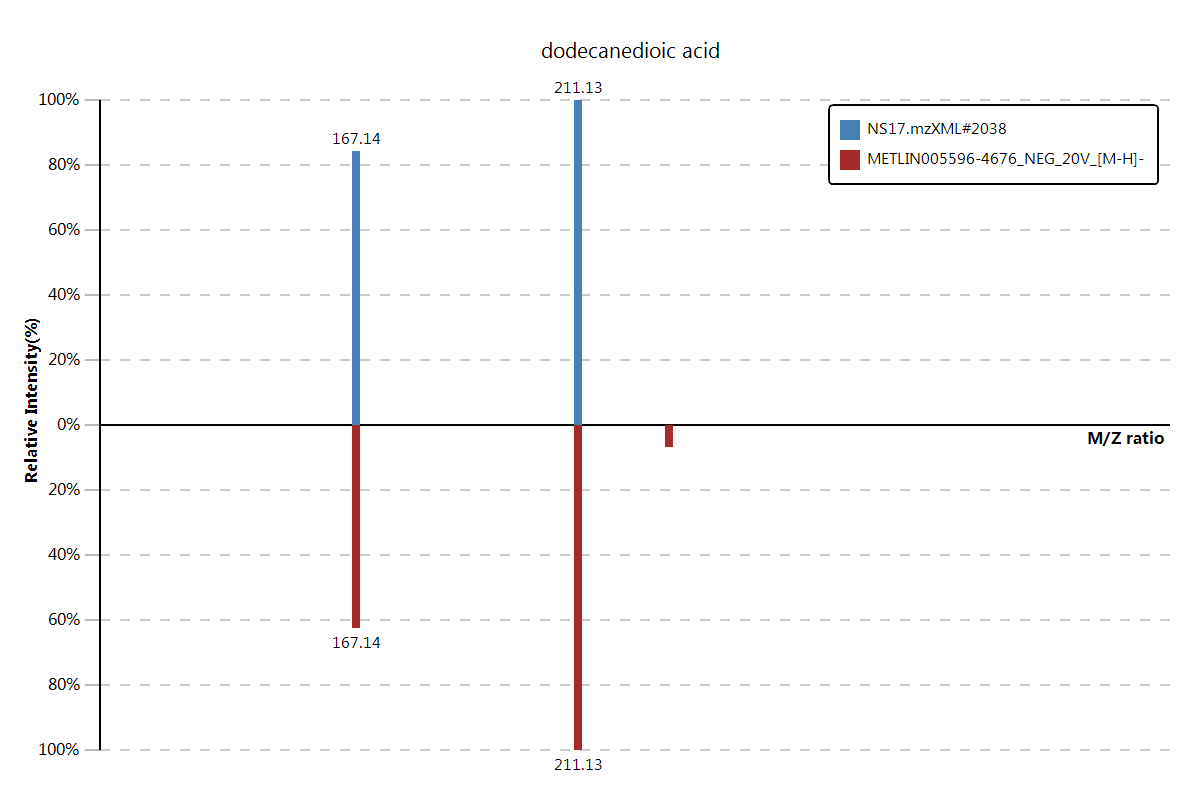


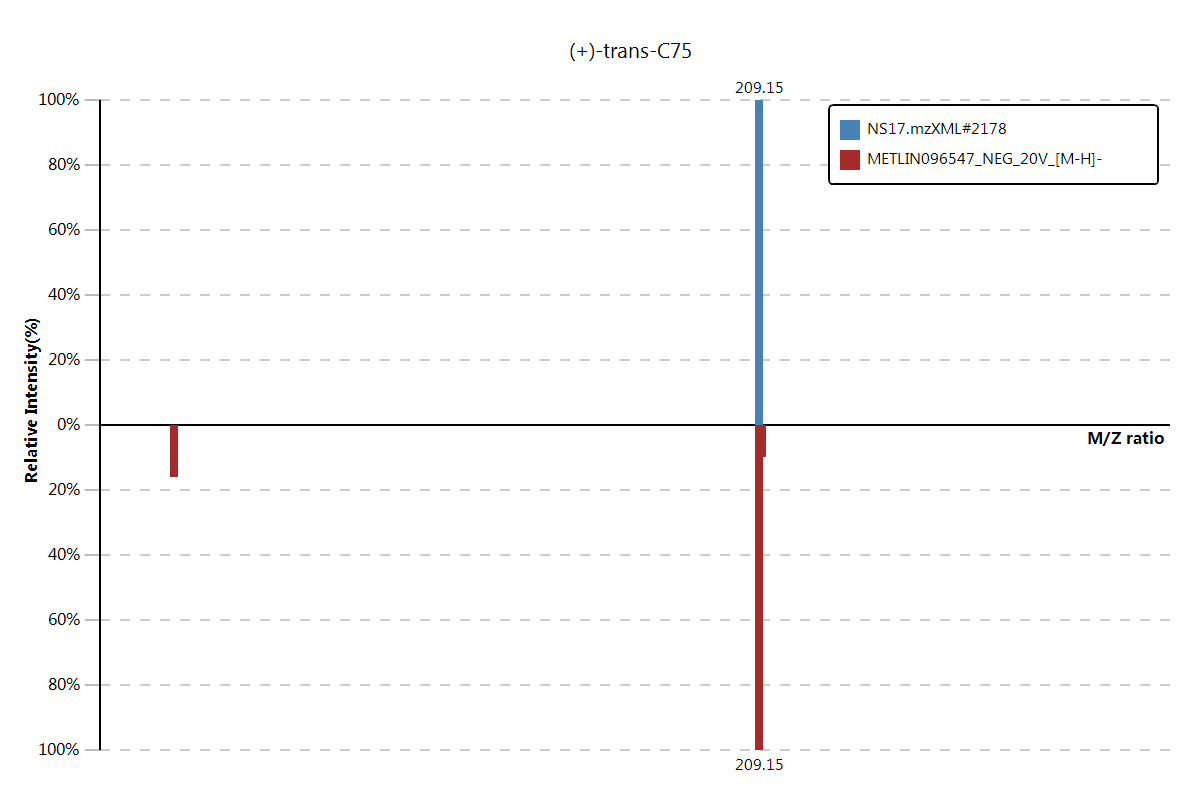


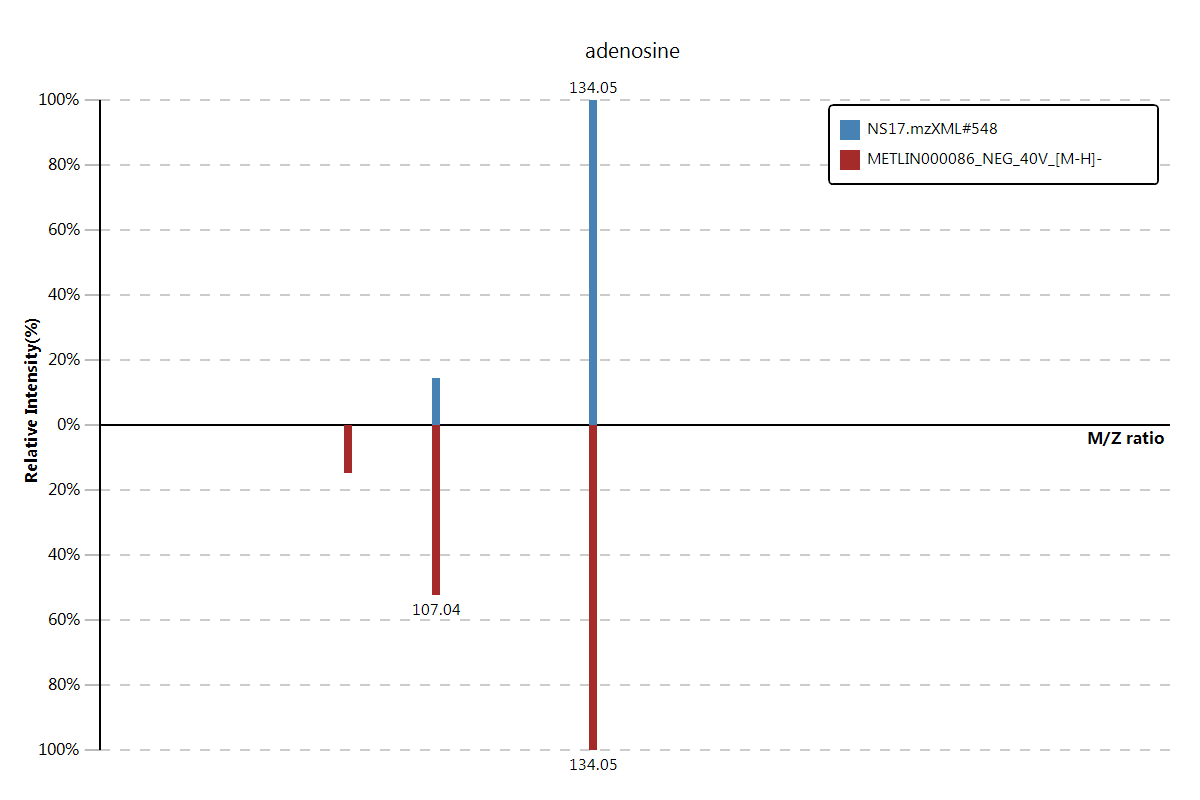


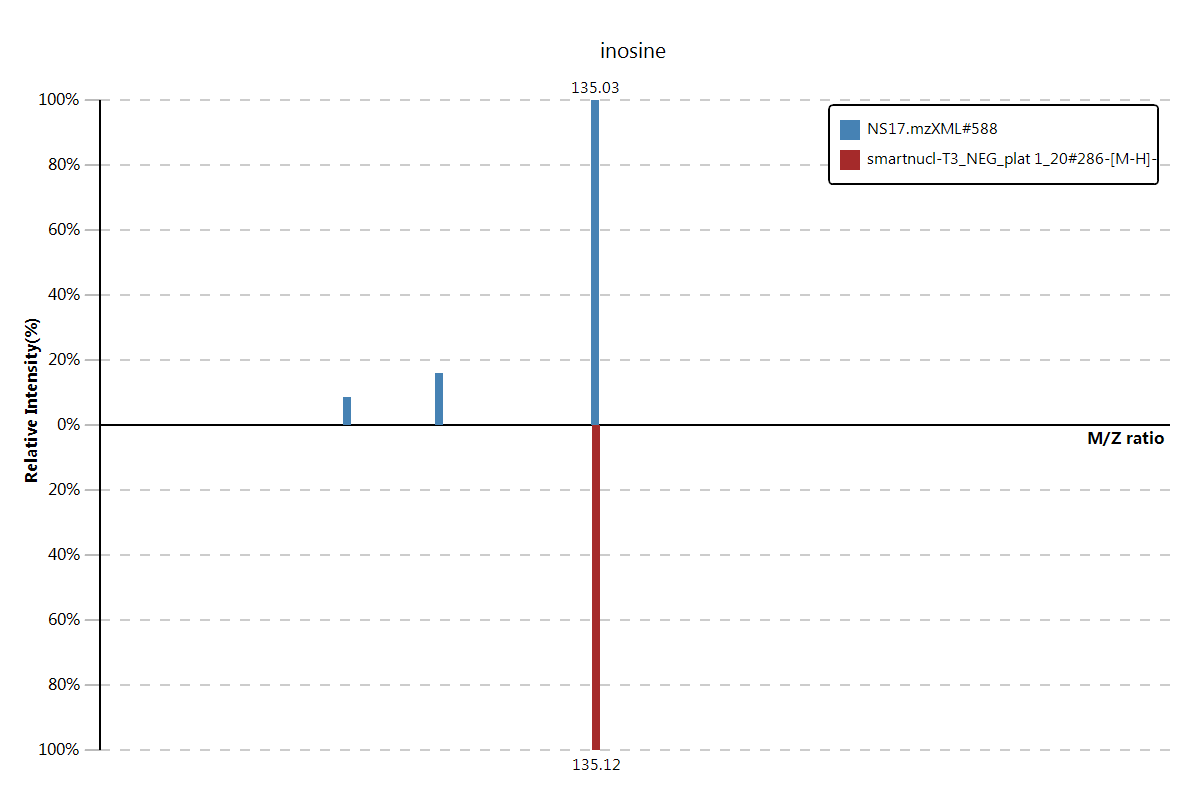


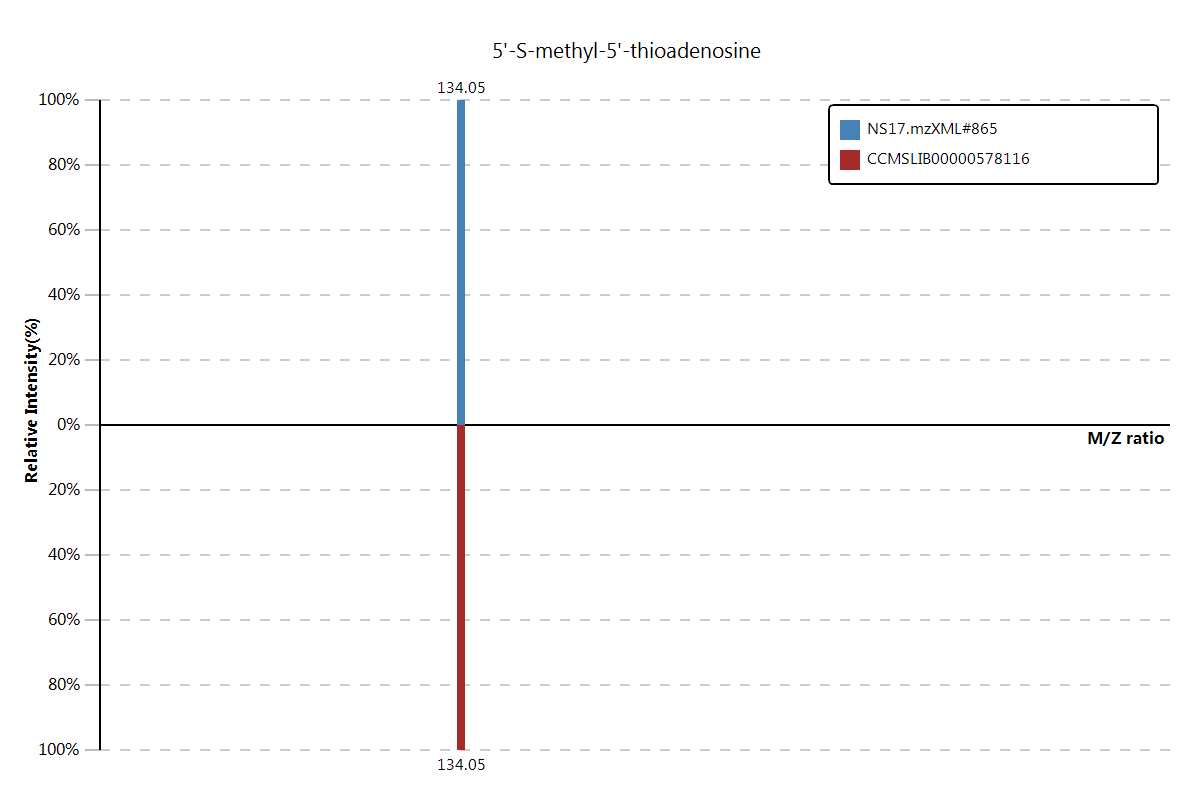


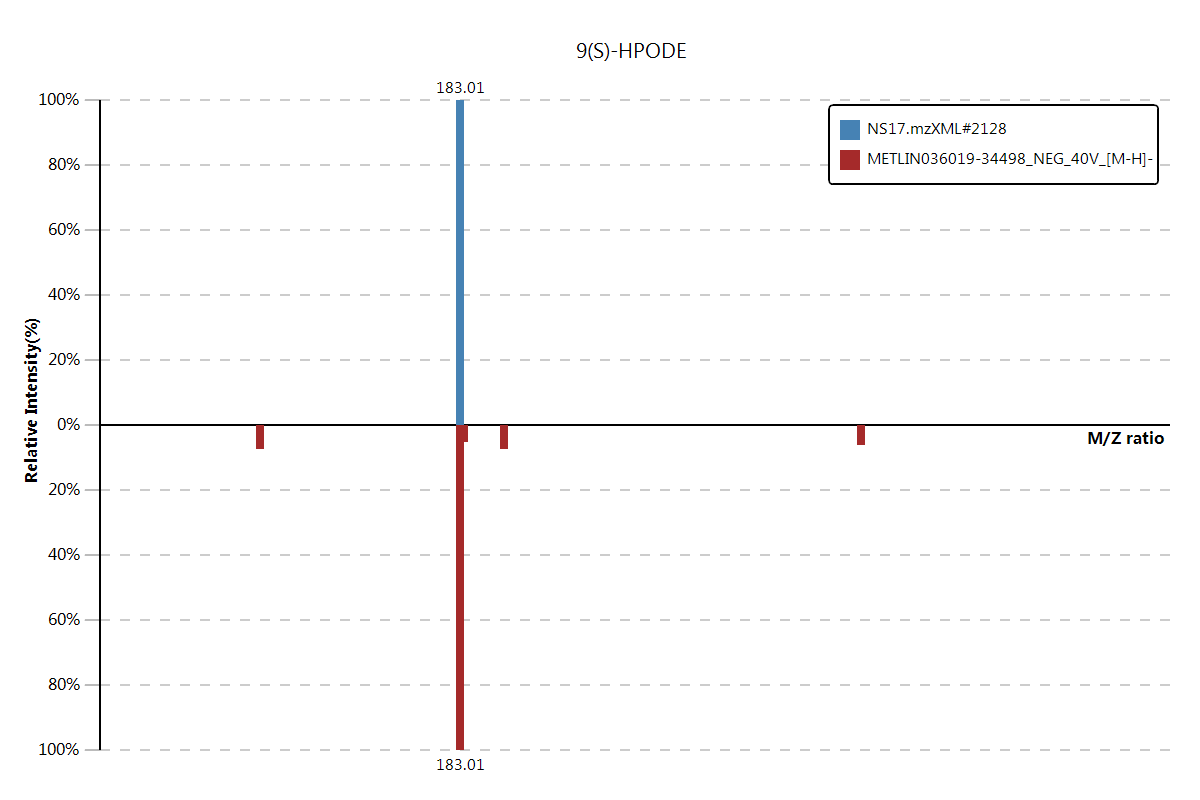


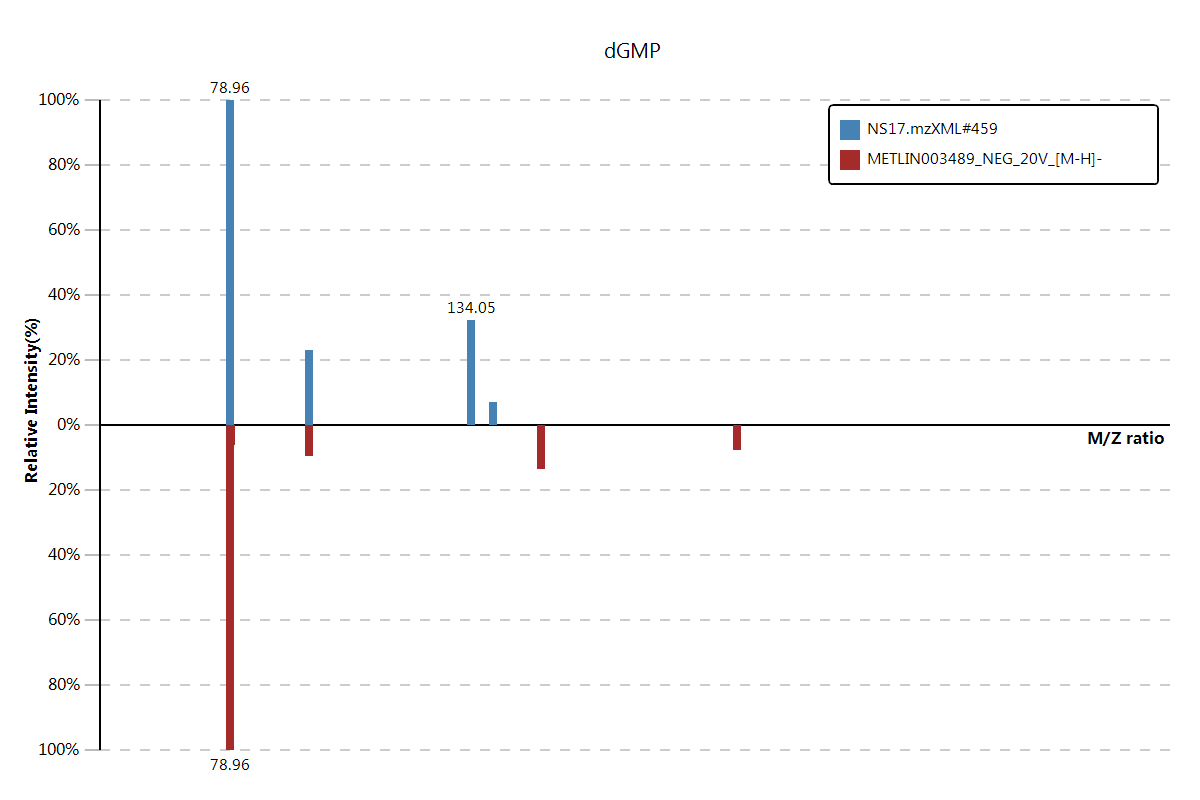


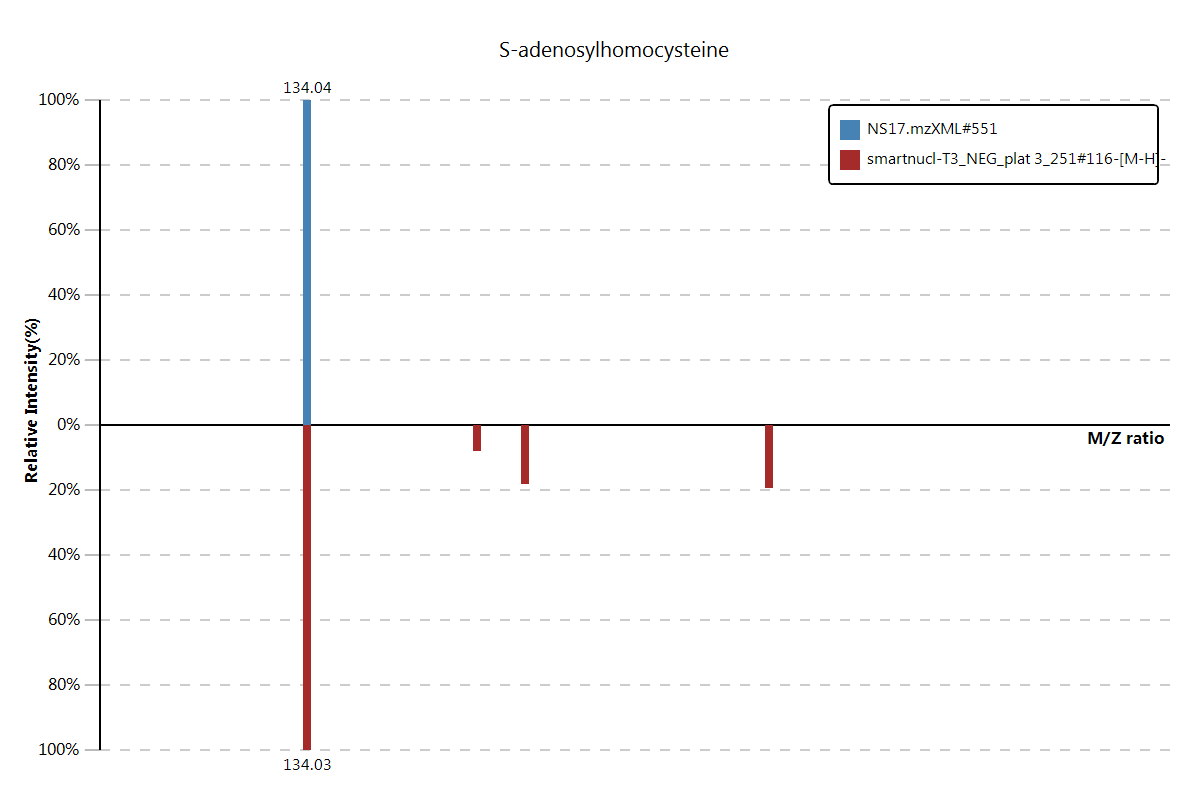


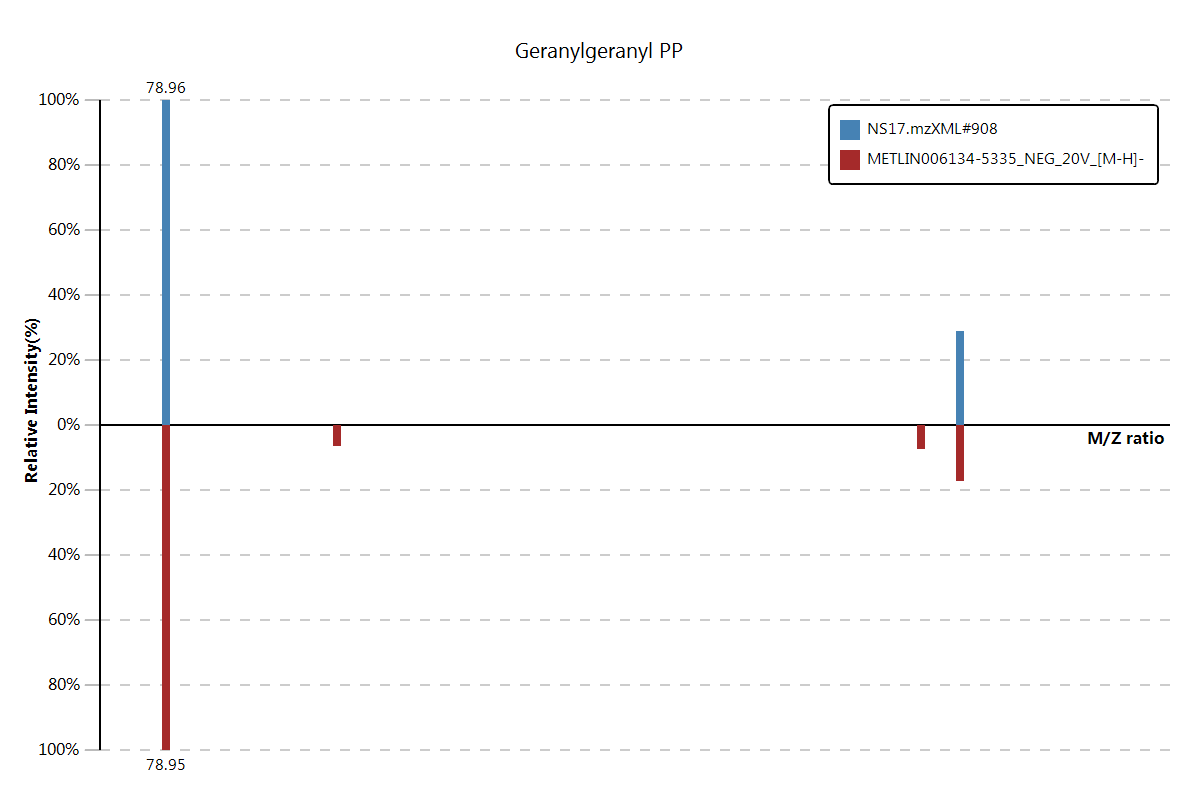


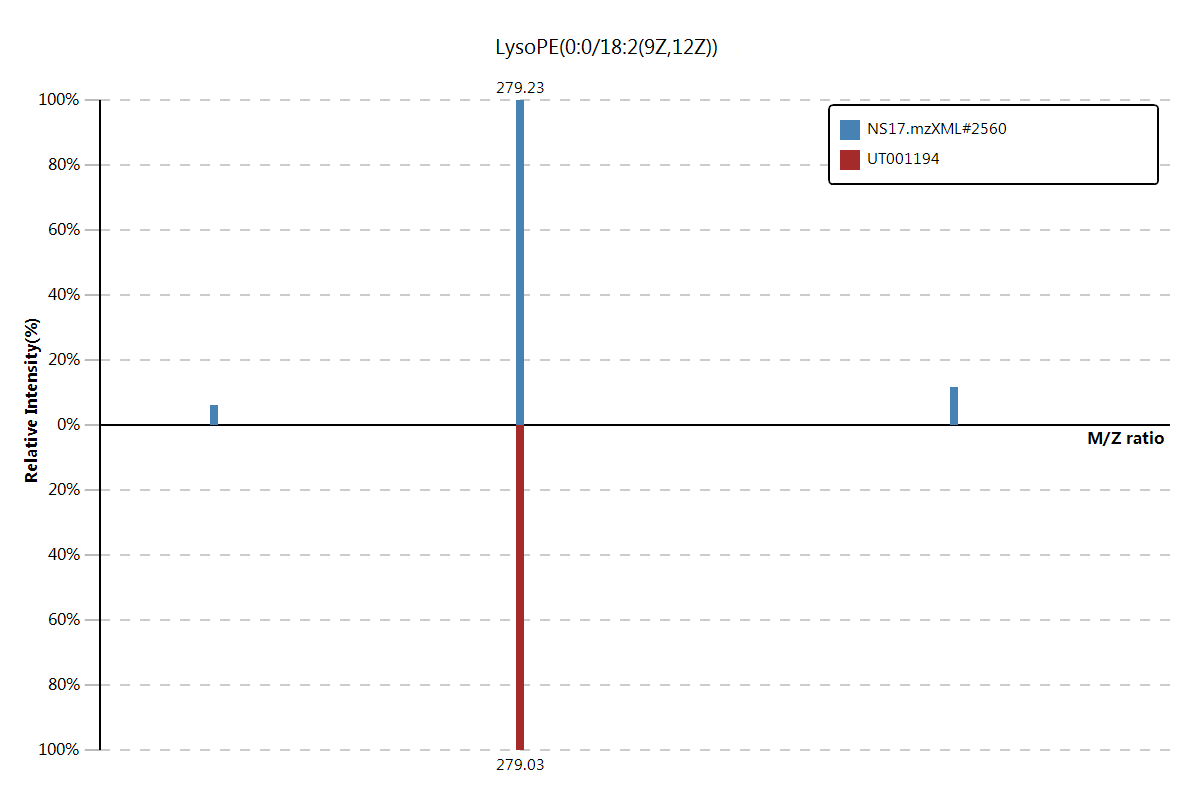


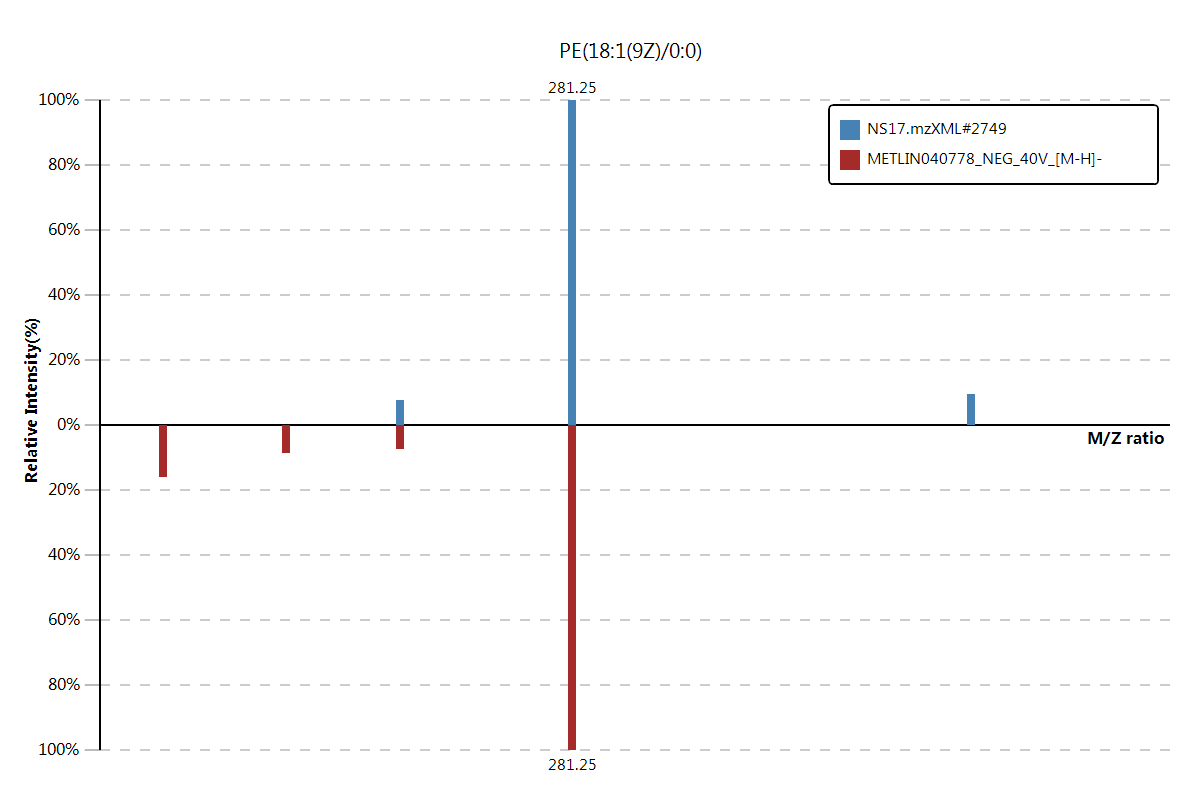


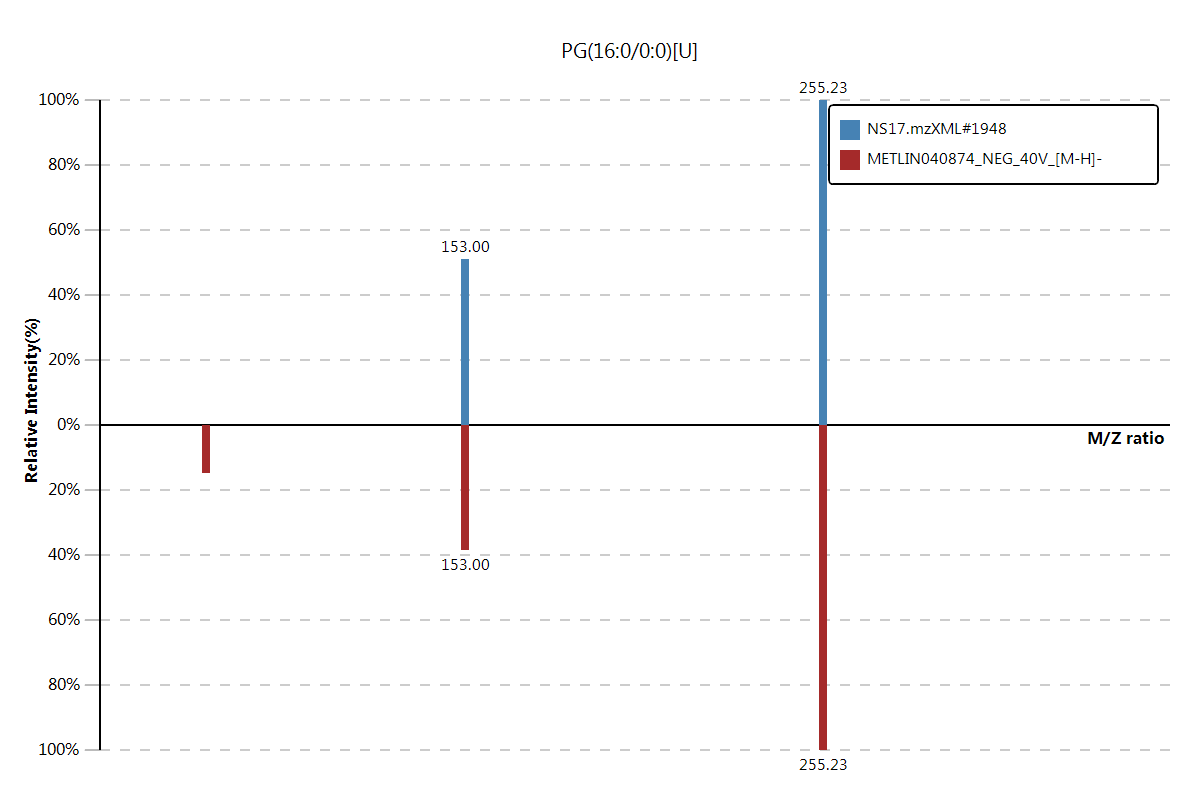


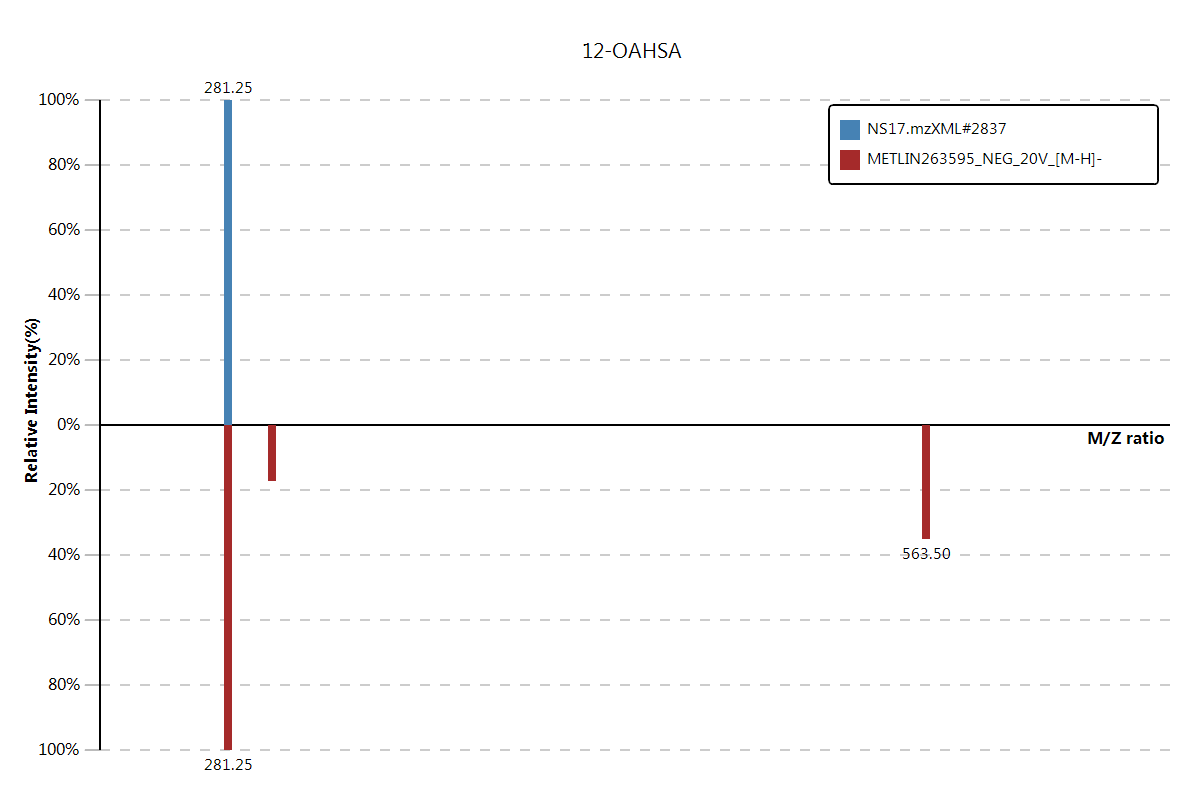

Supplement: Supplementary file 8 — Figure S1. Fragmentation spectrum for LC-MS. (DOCX 1592 kb) [file 12866_2019_1492_MOESM8_ESM.docx]
